# Platelet-derived biomaterial with hyaluronic acid alleviates temporal-mandibular joint osteoarthritis: clinical trial from dish to human

**DOI:** 10.1186/s12929-023-00962-y

**Published:** 2023-09-11

**Authors:** Bou-Yue Peng, Abhinay Kumar Singh, Ching-Yu Tsai, Chun-Hao Chan, Yue-Hua Deng, Chi-Ming Wu, Yen-Ru Chou, Wen Tsao, Chia-Yu Wu, Win-Ping Deng

**Affiliations:** 1 Department of Dentistry, Taipei Medical University Hospital, Taipei, 110301 Taiwan; 2 School of Dentistry, College of Oral Medicine, Taipei Medical University, Taipei, 110301 Taiwan; 3 Stem Cell Research Center, College of Oral Medicine, Taipei Medical University, Taipei, 110301 Taiwan; 4 Graduate Institute of Biomedical Materials and Tissue Engineering, College of Biomedical Engineering, Taipei Medical University, 110301 Taipei, Taiwan; 5 School of Oral Hygiene, College of Oral Medicine, Taipei Medical University, Taipei, 110301 Taiwan; 6 Division of Oral and Maxillofacial Surgery, Department of Dentistry, Taipei Medical University Hospital, Taipei, 110301 Taiwan; 7 Graduate Institute of Biomedical and Pharmaceutical Science, Fu Jen Catholic University, Taipei, 242062 Taiwan

**Keywords:** Platelet-derived biomaterial, TMJ-OA, Hyaluronic acid, Clinical trial

## Abstract

**Background:**

Bioactive materials have now raised considerable attention for the treatment of osteoarthritis (OA), such as knee OA, rheumatoid OA, and temporomandibular joint (TMJ) OA. TMJ-OA is a common disease associated with an imbalance of cartilage regeneration, tissue inflammation, and disability in mouth movement. Recently, biological materials or molecules have been developed for TMJ-OA therapy; however, ideal treatment is still lacking. In this study, we used the combination of a human platelet rich plasma with hyaluronic acid (hPRP/HA) for TMJ-OA therapy to perform a clinical trial in dish to humans.

**Method:**

Herein, hPRP was prepared, and the hPRP/HA combined concentration was optimized by MTT assay. For the clinical trial in dish, pro-inflammatory-induced in-vitro and in-vivo mimic 3D TMJ-OA models were created, and proliferation, gene expression, alcian blue staining, and IHC were used to evaluate chondrocyte regeneration. For the animal studies, complete Freund’s adjuvant (CFA) was used to induce the TMJ-OA rat model, and condyle and disc regeneration were investigated through MRI. For the clinical trial in humans, 12 patients with TMJ-OA who had disc displacement and pain were enrolled. The disc displacement and pain at baseline and six months were measured by MRI, and clinical assessment, respectively.

**Results:**

Combined hPRP/HA treatment ameliorated the proinflammatory-induced TMJ-OA model and promoted chondrocyte proliferation by activating SOX9, collagen type I/II, and aggrecan. TMJ-OA pathology–related inflammatory factors were efficiently downregulated with hPRP/HA treatment. Moreover, condylar cartilage was regenerated by hPRP/HA treatment in a proinflammatory-induced 3D neocartilage TMJ-OA-like model. During the animal studies, hPRP/HA treatment strongly repaired the condyle and disc in a CFA-induced TMJ-OA rat model. Furthermore, we performed a clinical trial in humans, and the MRI data demonstrated that after 6 months of treatment, hPRP/HA regenerated the condylar cartilage, reduced disc displacement, alleviated pain, and increased the maximum mouth opening (MMO). Overall, clinical trials in dish to human results revealed that hPRP/HA promoted cartilage regeneration, inhibited inflammation, reduced pain, and increased joint function in TMJ-OA.

**Conclusion:**

Conclusively, this study highlighted the therapeutic potential of the hPRP and HA combination for TMJ-OA therapy, with detailed evidence from bench to bedside.

*Trial registration* Taipei Medical University Hospital (TMU-JIRB No. N201711041). Registered 24 November 2017. https://tmujcrc.tmu.edu.tw/inquiry_general.php.

**Supplementary Information:**

The online version contains supplementary material available at 10.1186/s12929-023-00962-y.

## Introduction

Temporomandibular joint osteoarthritis (TMJ-OA) is the most common degenerative disease affecting women more commonly than men (female to male: 2:1) [1, 2]. TMJ-OA is characterized by chronic pain, cartilage degradation, and subchondral bone erosion. The TMJ is a complex joint comprising the mandibular condyle, temporal bone, and articular disk and plays essential roles in chewing and speaking [3]. The TMJ condyle surface is composed of fibrocartilage, and upon prolonged lesion or inflammatory conditions, the cartilage undergoes decreased matrix deposition and homeostasis dysfunction, requiring spontaneous healing [4]. Currently, treatments for TMJ-OA mainly focused on symptomatic therapies to manage pain relief, however, effective therapy to repair and regenerate damaged TMJ is still lacking.

Human platelet-rich plasma (hPRP), a blood derived biomaterial which mainly composed of platelets, proteins, and also contains some white blood cells (WBCs) [5, 6]. The therapeutic effect of hPRP are mediated by activation of platelets releases a cocktail of several growth factors that act as biomaterials, including platelet-derived growth factor (PDGF), vascular growth factor (VGF), endothelial growth factor (EGF), and transforming growth factor (TGFβ1); and can promote chondrocyte regeneration [7]. Recently, we have demonstrated that hPRP enhanced cerebro- and renoprotective activities in late stage cerebrorenal syndrome through anti-inflammatory, and anti-oxidant activities in a rat model. In our previous studies, we have also shown that hPRP delays aging through reprogramming the stem cell from senescence, and this study also demonstrated the osteogenic potential of hPRP. hPRP has recently been used for treating knee OA because it reduces pain and promotes cartilage repair and joint function [8–10]. In addition, hyaluronic acid (HA) is an essential bioactive material of the cartilaginous matrix and a key regulator of chondrocyte function [11]. HA is a naturally derived non-sulfated glycosaminoglycan, nonfibrillar component that is nonimmunogenic, and is found in most human joints and connective tissues [12]. HA physiologically occurs within the synovial fluid, which helps to maintain the mechanical load, fibrocartilage and chondrocytes function [13]. In aging, hyaluronidase is activated and degrades HA, leading to condylar cartilage degradation and inflammation in TMJ [14]. In recent years, researchers have expressed interest in the use of naturally derived biomaterials, including hPRP and HA, for treating OA [15]. However, several studies have used hPRP, HA, or their combination for reducing pain and inflammation only, but few have investigated the regenerative potential of hPRP and HA combination for TMJ-OA.

Moreover, hPRP or HA or combined treatment exerted therapeutic efficacies against chronic pain reduction and cartilage and bone regeneration in different types of OA [16, 17]. Recently, a study used the HA and PRP combination for the in-vivo regeneration of the articular disc in TMJ, but the researchers did not show anti-inflammatory or OA-like conditions and also not evaluate cartilage and bone regeneration [18]. Most studies have supported the clinical use of the PRP and HA combination for treating knee OA, and the results indicate that their combination provides better clinical improvement than PRP or HA monotherapy [17, 19, 20]. However, their relevance to cartilage regeneration and disc displacement in TMJ-OA is unclear.

Therefore, in this study, we evaluated the therapeutic potential of combined hPRP/HA clinical trials in dish to human in TMJ-OA. In particular, hPRP with a higher number/purity of platelets was prepared. The hPRP/HA clinical trial in dish (in-vitro and 3D model) were performed on pro-inflammatory-induced TMJ-OA model. For the clinical trial in animals, CFA was used to induce TMJ-OA in rats for examining the anti-inflammatory, condyle regeneration, articular disc area reduction, and subchondral bone sclerosis inhibition potential of the hPRP/HA combination, and the underlying mechanism was also investigated. Furthermore, we enrolled the TMJ-OA patients for clinical trial of hPRP/HA to examine the clinical and pathological features of TMJ OA, such as chronic pain, maximum mouth opening (MMO), disc displacement, and condyle regeneration. Our results would provide insights into the therapeutic potential of hPRP/HA treatment for TMJ-OA therapy, with evidence from bench to bedside.

## Materials and methods

### Isolation and culture of TMJ condylar chondrocytes

TMJ chondrocytes were isolated from 8-week-old Sprague Dawley rats. Briefly, condylar cartilage tissues were dissected and incubated with 0.25% trypsin (Sigma-Aldrich, St. Louis, USA) at 37 °C for 15 min, and bone and muscle tissues were removed. Subsequently, isolated chondrocytes from TMJ tissues were digested with 1 mg/mL collagenase type II (Coll-II; Sigma-Aldrich, USA) at 37 °C for 3 h. TMJ chondrocytes were seeded at 5 × 10^3^ cells/cm^3^ in alpha-minimum essential medium (Gibco, Taiwan) supplemented with 10% fetal bovine serum until all chondrocytes reached confluence for 21 days.

### In-vitro TMJ OA model

To establish an in-vitro TMJ-OA model, TMJ chondrocytes were cultured with proinflammatory cytokines, including IL-1β and tumor necrosis factor (TNF)-α, to induce OA inflammation, as previously described [21]. For cell attachment, the cells were cultured in DMEM/F12 medium with a combination of 20 ng/mL IL-1β (Sigma-Aldrich) and 40 ng/mL TNF-α (Sigma-Aldrich, USA).

### hPRP and HA preparation

hPRP was prepared and quantified as described previously [22]. The HA (MW: 50–120 kD)–conditioned medium was prepared as described previously [21]. To prepare hPRP, we used the patented, specially designed platelet concentrate separator containing ACD-A as an anticoagulant and a specific separator gel for preparation of hPRP, and this process prevented contamination of blood components, including red blood cells (RBC) and leukocytes. Briefly, 5–7 mL of human peripheral blood was collected into a PLTenus PLUS Platelet Concentrate Separator (TCM Biotech International Corp., Taiwan) through a sterile venipuncture. Further, the collected blood was centrifuged at 500–1200G for 8 min. The 4 mL of mixed plasma and platelets that remained above the gel layer were harvested and then collected in a falcon tube until use. The origin of HA were from animal, and clinically approved for human use. ARTZ-Dispo HA (Seikagaku) was used for HA conditioned media in DMEM/F12 with 1% FBS (for basal cell maintenance) and has a weight-average molecular weight (MW) of 50–120 kDa. The optimal HA and hPRP concentrations were prepared using various maintain condition cell culture media, and their efficacies were determined.

### Cell viability and proliferation assays

MTT [(3-(4, 5-dimethylthiazol-2-yl)-2, 5-diphenyl tetrazolium bromide) assay with tetrazolium salt reagent (Roche) was performed to determine the effects of HA and hPRP on the viability of TMJ chondrocytes. The cells were seeded into a 96-well plate at a density of 2 × 10^4^ cells/well and were treated with different concentrations of HA (0–500 mg/mL) or hPRP (TGF-β1, 100 pg/mL to 1 ng/mL)–conditioned medium, whereas 1% fetal bovine serum was used as the control. Qualitative cellular viability analysis was performed on days 1, 3, 5, and 7 using the time-course MTT assay, and absorbance was measured by using Multiskan PC (Thermo Labsystem). Next, TMJ chondrocytes were treated with proinflammatory cytokines for 2 days and were then treated with the HA and hPRP combination or monotherapy. The cells were then harvested, and cell proliferation was examined by measuring the cell count on an automated cell counter (Life Technologies).

### Semi-quantitative reverse-transcription PCR

RNA was isolated from TMJ chondrocytes using TRIzol reagent (Invitrogen Life Technologies). Gene expression levels were measured using RT-PCR. The PCR primers are listed in Table 1. PCR products were separated through electrophoresis on 2% agarose gels (Agarose I; AMRESCO), with SYBR safe (BIOTOOLS) staining, and the images were analyzed using Mutigel-21 (Fluorescent Gel Image System TOP BIO).

**Table 1:** Primer used in this study

Gene	Primer sequences (5’ → 3’)	Product size (bp)
Interlukin 1β	CACCTCTCAAGCAGAGCACAG GGGTTCCATGGTGAAGTCAAC	79
COX2	TGCGATGCTCTTCCGAGCTGTGCT TCAGGAAGTTCCTTATTTCCTTTC	480
MMP3	ACCTATTCCTGGTTGCTG GGTCTGTGGAGGACTTGTA	105
MMP13	CTGACCTGGGATTTCCAAAA ACACGTGGTTCCCTGAGAAG	96
SOX9	CGTCAACGGCTCCAGCA TGCGCCCACACCATGA	69
Col I	CTTCGTGTAAACTCCCTCCATCC AAGTCCATGTGAAATTGTCTCCCA	136
Col II	GAGTGGAAGAGCGGAGACTACTG CTCCATGTTGCAGAAGACTTTCA	81
Aggrecan	CTAGCTGCTTAGCAGGGATAACG TGACCCGCAGAGTCACAAAG	108
GAPDH	CGGATTTGGCCGTATCGG CAATGTCCACTTTGTCACAAGAGAA	65

### In-vivo mimic TMJ-OA 3D model

Neocartilage samples of TMJ chondrocytes were combined with collagen and cultured in a rotatory cell culture system (RCCS, Synthecon) for 4 weeks, as described previously [23]. The samples were subjected to various treatments: DMEM/F12 (control group), 20 ng/mL IL-1β and 40 ng/mL TNF-α (for arthritic neocartilage formation), or 250 mg/mL HA and 1 ng/mL hPRP (treatment group)–conditioned medium in a 37 °C, 5% CO_2_ incubator; all media were changed every 2 days. After 4 weeks of culture, the neocartilage samples were collected and subjected to hematoxylin and eosin (H&E) staining, immunohistochemical (IHC) staining of Col-II (MAB1330, Chemicon International), and Alcian blue staining for proteoglycan to determine chondrogenic ECM accumulation.

### Histologic and IHC staining

All cells were fixed with 4% paraformaldehyde for 10 min and subjected to Alcian blue staining and immunocytochemistry staining. TMJ chondrocytes were stained with Alcian blue solution (Sigma-Aldrich) for 30 min. The cells were also subjected to IHC staining, as described previously [21]; the primary antibody against Col-II was used to analyze chondrogenic regeneration. All images were acquired using DPC controller software (Olympus).

### TMJ-OA animal model

Eight-week-old female Sprague Dawley rats weighing 200–300 g were purchased from Bio-LASCO Taiwan. The experiment protocol was approved by the Institutional Animal Care and Use Committee of Taipei Medical University (Approval Number- LAC-2017-0147). The rats were maintained in the animal room under the following conditions: 25 °C and 50% relative humidity. The TMJ-OA model was induced by CFA injection. Briefly, 50 μL CFA (0.01 mg/mL) (Sigma-Aldrich, USA) was injected into the right side anterosuperior compartment of the TMJ on Day 0 (1st injection) and Day 14 (2nd injection). Two weeks after TMJ-OA induction, the rats received HA (250 mg/mL) and PRP injections (1 ng/mL), and the control group received the same volume of saline injections. All animals were examined for body weight and head width and were subjected to magnetic resonance imaging (MRI).

### MRI evaluation

At 2 weeks and 1 week before treatment, the animals were anesthetized and analyzed with a 7-T MRI scanner (Bruker Pharmascan) to evaluate TMJ OA induction. At 3 and 4 weeks after treatment, MRI was performed again to evaluate the effect of HA + hPRP. Additionally, at 4 weeks, joint samples were collected from all groups and evaluated with MRI (inner radius: 9 cm, gradient magnetic field: 380 mT/m). Next, all bone tissues were collected and imaged using a SkyScan-1076 Micro-CT system (Skyscan, Belgium) for measuring the percentage of bone volume over total volume (BV/TV, %), trabecular thickness (Tb·Th, mm), trabecular separation (Tb·Sp, mm), and trabecular number (Tb·N, 1/mm).

### Histological and immunohistological staining

TMJ tissue samples were collected, fixed in 10% formalin, and processed. Next, 10-μm-thick TMJ sections were stained with H&E staining and Alcian blue solution (Sigma-Aldrich) for 30 min. IHC staining was also conducted, as previously described. The primary antibody against Col-II (MAB1330, Chemicon International) was used to determine chondrogenic ECM accumulation in TMJ tissues. Each group contained six animals.

### Clinical study of HA and PRP therapy

The clinical study was approved by the Taipei Medical University Hospital (TMU-JIRB No. N201711041). 20 patients of TMJ-OA enrolled in the clinical trial were randomized to two groups:- 10 patients in PRP/ HA (1 ng/mL/250 μg/mL) treatment group, and 10 patients in non-treatment (control/disease) group, and arthrocentesis was used for the control group. All patients was examined by MRI imaging, and scores of MMO, visual analogue scale (VAS), and activity of daily life (ADL). For the VAS score, we give the oral analgesic for pain control in both groups. Inclusion criteria for participants included the following: ethnically Taiwanese, male and female, age between 30 and 60 years, VAS score > 4, MMO score < 3.5, and ADL score < 20. Exclusion criteria included any of the following: (1) poorer management of diabetes (2) lactating or pregnant women, (3) Severe renal, liver or heart disease and (4) with positive HBs antigen, positive HCV antibody, positive HIV antibody, positive HTLV antibody, after data collection and all patients gave written consent by signing informed consent form. We also performed the sensitivity analysis of our clinical trial by last observation carried forward (LOCF) imputation method for VAS score (Additional file 1: Tables S2, and S3.1), MMO score (Additional file 1: Tables S4, and S5.1), and ADL score (Additional file 1: Tables S6, and S7.1).

### Statistical analysis

Quantitative data were expressed as means ± SD. Statistical analysis was adequately performed by ANOVA followed by Bonferroni multiple-comparison post hoc test. SAS statistical software for Windows version 8.2 (SAS institute, Cary, NC) was utilized. A probability value < 0.05 was considered statistically significant.

## Results

**Fig. 1:**
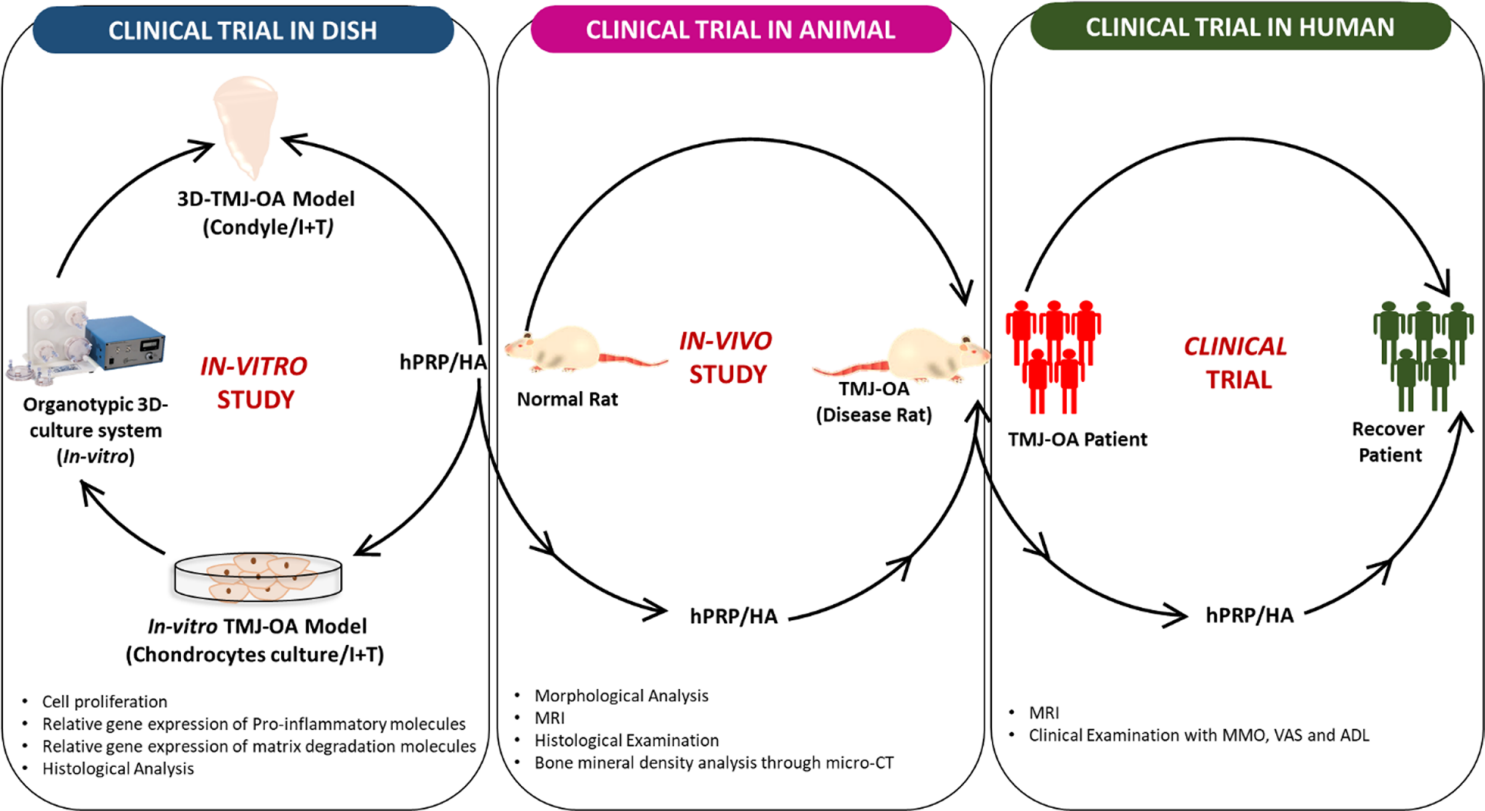
Schematic of hPRP/HA treatment in TMJ-OA disease from bench to beside

In the current study, we extracted hPRP from human blood and investigated the combined treatment of hPRP and HA on the recovery of TMJ-OA from clinical trials in dish to humans. First, for clinical trial in dish, chondrocytes isolated from rat TMJ were used as an in-vitro (2D and 3D)-TMJ-OA model which was created by pro-inflammatory cytokines, namely IL-1β + TNF-α, designated as I + T. Moreover, a 3D TMJ-OA model was also established, in which a condyle-like structure was formed using collagen scaffold–encapsulated culture under the I + T condition. Second, for pre-clinical trials in animals, an in-vivo TMJ-OA rat model was created by CFA administration to evaluate condylar degeneration. Finally, we recruited patients with TMJ OA for the human trial. Thus, the effects of combine therapy of hPRP/HA on TMJ-OA recovery was intensively examined as summarized in Fig. 1.

### Effect of hPRP/HA therapy on proliferation of chondrocytes in TMJ-OA in-vitro 2D mode

**Fig. 2:**
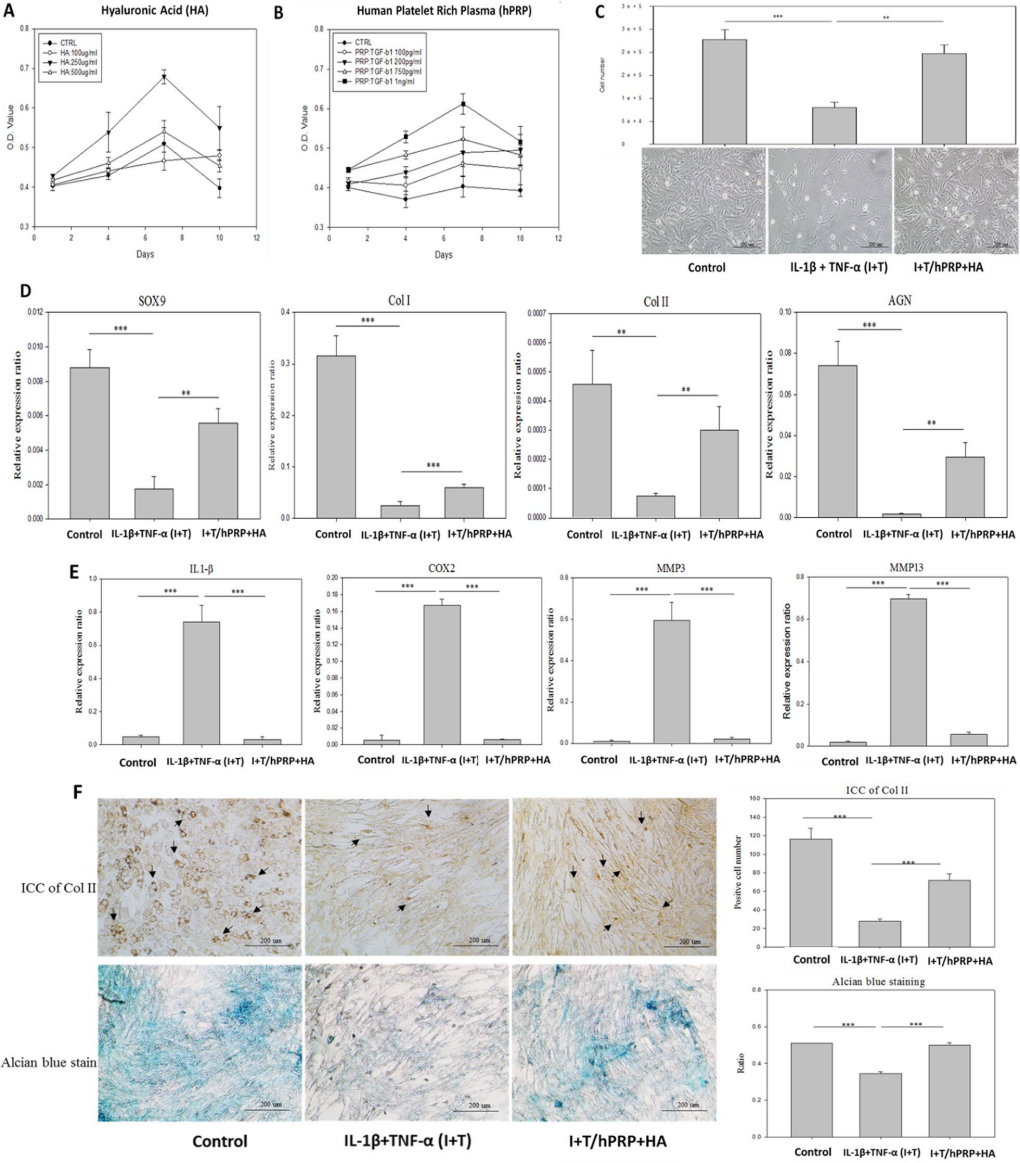
In-vitro 2D hyaluronic acid (HA) and human platelet-rich plasma (hPRP) promote cell activity and anti-inflammatory effects of rat TMJ chondrocyte. Optimal concentration of (**A**) HA and (**B**) hPRP dosage in rat TMJ chondrocytes that were analyzed using the MTT assay. **C** The effect of HA and PRP on the cell numbers and morphological changes (200 ×) of rat TMJ chondrocytes after 2-day treatment with IL1β + TNF-α (I + T)-conditioned medium. I (10 ng/mL) + T (20 ng/mL) were added to the medium to create an in-vitro proinflammatory cytokine-induced arthritic cell model. Real-time PCR analyses of monolayer cultures of rat TMJ chondrocytes after 2-day treatment with I + T-conditioned medium. The results revealed that the combination of HA and hPRP (**D**) significantly increased cartilage-specific gene expression, including SOX9, collagen type I (Col-I), collagen type II (Col-II), and aggrecan, and **E** significantly inhibited I + T stimulated inflammatory genes including IL-1β, COX2, MMP-3, and MMP-13. **F** Immunocytochemistry of Col-II (black arrow indicates presence of Col-II) and its quantification (upper panel) and Alcian blue stain and its quantification (lower panel). *p < 0.05, **p < 0.01, and ***p < 0.001 compared with I + T and control group using paired t test. The results are shown as mean ± SD for three replicate

To evaluate the optimal HA and hPRP concentrations on rat TMJ chondrocytes, isolated chondrocytes from TMJ were treated with increasing concentrations of HA or hPRP. The dose for most viability of rat TMJ chondrocytes was 250 μg/mL for HA and 1 ng/mL for hPRP (Fig. 2A, B). Thus, MTT assay results indicated that 250 μg/mL HA and 1 ng/mL hPRP were deemed as their optimal concentrations for further tests of the hPRP/HA therapy. A cellular TMJ-OA 2D model was constructed with IL-1β + TNF-α (I + T) to induce inflammation in rat TMJ chondrocytes to mimic the in-vivo system. The chondrocytes were cultured in the (I + T)-conditioned medium and then treated with or without hPRP/HA for 48 h. Under the I + T condition, the cell number showed a significant decrease, and fibroblast-like morphology was restored maximally by combined hPRP/HA treatment, and aggregate and polygonal cell morphology was found (Fig. 2C; Additional file 1: Fig. S1).

### Combined hPRP/HA therapy modulates gene expression changes in the TMJ-OA in-vitro model

The therapeutic effects of the hPRP/HA treatment were assessed through gene expression analysis of the rat TMJ chondrocytes harvested at 48-h post treatment with I + T. The genes involved in the chondrogenesis process were analyzed. The expression of chondrogenic genes, such as SOX9, Col-II, Col-I, and aggrecan, was significantly decreased, which was restored after treatment with the hPRP/HA (Fig. 2D). Notably, hPRP/HA treated chondrocytes showed significantly reduced expression of proinflammatory genes (IL-1β, COX2, and MMP3) and matrix regulation genes (MMP13); the expression of these genes were enhanced by I + T (Fig. 2E), indicating that the hPRP/HA decreased the levels of activated inflammatory and matrix inhibitory molecules to enhance chondrogenesis. Furthermore, the accumulation of proteoglycan and Col-II in the chondrogenic matrix was determined through Alcian blue and ICC staining, respectively, along with their quantification (Fig. 2F). In the treatment group, the hPRP/HA modulated inflammatory–depleted proteoglycan levels in the cellular OA model, indicating that combined hPRP/HA treatment enhanced the positive signal of Col-II, specifically in the proliferative area (marked with an arrow). However, the group without hPRP/HA treatment exhibited complete degradation of Col-II and proteoglycan compared with the control group.

### Combined hPRP/HA therapy promotes chondrocyte regeneration in TMJ-OA in-vitro 3D condyle model

**Fig. 3:**
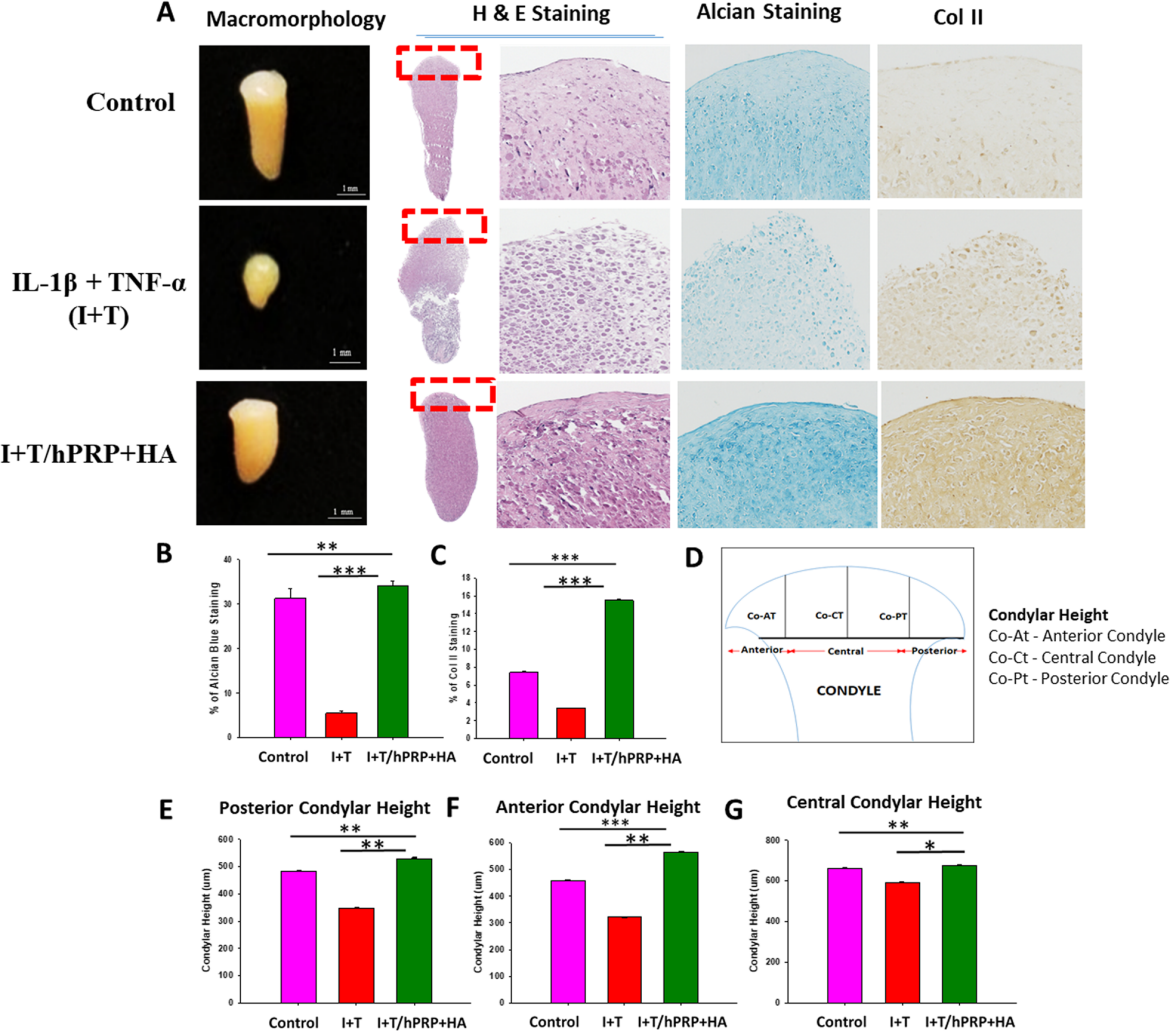
In-vitro 3D hyaluronic acid (HA) and platelet-rich plasma (hPRP) promote cell activity of rat TMJ chondrocytes. For the TMJ-OA model, neocartilage of TMJ chondrocytes/collagen constructs were cultured for 4 weeks in basal control, I + T, and I + T/hPRP/HA -conditioned medium. Constructs were then histologically examined using (**A**) macromorphology, hematoxylin and eosin (H&E) staining, IHC staining for Col-II, and Alcian blue staining. (**B**) Percentage areal deposition of s-GAG by quantification of TB-stained area, and (**C**) percentages of Col-II deposition in TMJ condylar cartilage. (**D**) Schematic of the anterior, central, and posterior regions of the rat TMJ condylar head. Results (**E**) posterior, (**F**) anterior, and (**G**) central condylar height. *p < 0.05, **p < 0.01, and ***p < 0.001 compared with I + T and control group using paired t test. The results are shown as mean ± SD for three replicate

The results from the cellular TMJ-OA 2D model indicated that combined hPRP/HA treatment could strongly modulate inflammation and chondrogenesis to enhance regeneration potential and rescue chondrocyte degradation. To further validate the effects of combined hPRP/HA treatment in an in-vivo mimic microenvironment condition, rat TMJ chondrocyte encapsulated in collagen-derived scaffold of 3D construct with rotary stem cell technique. In our previous studies, we used a cartilage matrix for direct chondrogenesis in a 3D rotary stem cell system [21]. The construct resembles the condyle, with morphological changes observed after 4 weeks of cultivation. The 3D condyle cultured with I + T showed significantly deformed macromorphology, less condylar height, degraded cartilage-like outer surface, and proteoglycan deposition as compared with the control. However, after treatment with the hPRP/HA, the construct exhibited an elastic macromorphology indicating that cell proliferation increased to regenerate chondrocytes to form a condyle, with a smoother cartilage outer surface, improvement in matrix deposition, and condylar height. By contrast, H&E staining revealed higher cell death in the I + T group (Fig. 3A). Similarly, as in the in-vitro model, we conducted Alcian blue and Col-II IHC staining to examine the effect of the hPRP/HA on proteoglycan and Col-II deposition in the chondrogenic matrix, respectively. The results revealed that hPRP/HA-treated 3D condyle cartilage displayed matrix restoration with high proteoglycan deposition (Fig. 3B) and Col-II (Fig. 3C). The hPRP/HA-treated condyle also showed in the anterior, posterior, and central regions of condylar heights (Fig. 3D–G).

Collectively, the results for the in-vitro 2D and 3D TMJ-OA disease models demonstrated that combined hPRP/HA treatment promoted TMJ chondrocyte regeneration, inhibited the degeneration of condylar cartilage by reducing proinflammatory factors and matrix regulating factor, and enhanced the expression of the genes for chondrocyte regeneration and matrix restoration of condylar cartilage.

### Combined hPRP/HA therapy in TMJ-OA induced animal model

**Fig. 4:**
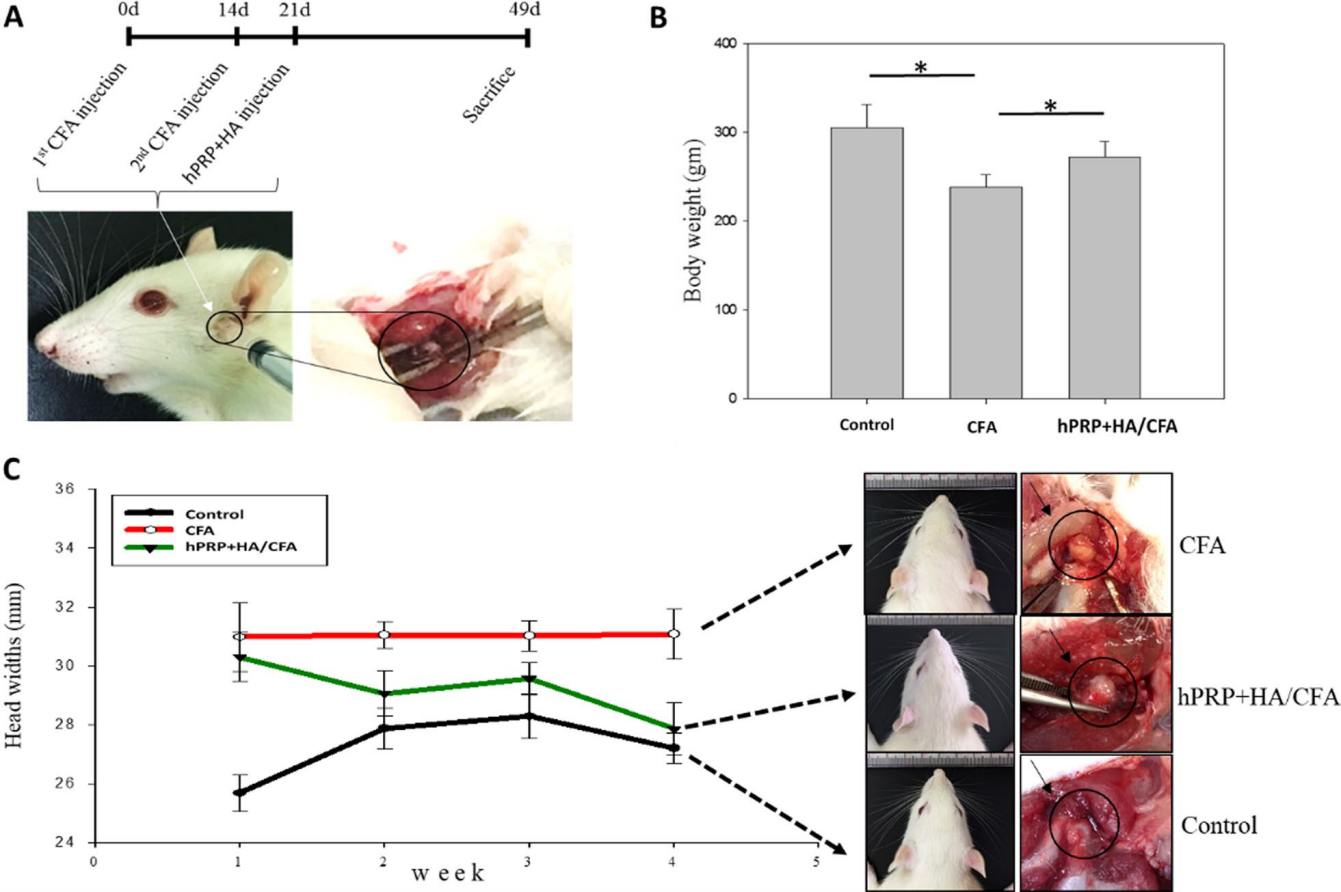
Experimental design and behavioral evaluation of combined hPRP/HA treatment in CFA-induced TMJ osteoarthritis. **A** Time course and experimental design. TMJ-OA was induced by two injections of CFA at 0 and 14th day, followed by combined hPRP/HA treatment in the 21st day. CFA and hPRP/HA were administered through the lateral puncture technique. **B** Body weight changes after treatment with hPRP/HA. **C** Representative photographs demonstrate that the head width of rats is increased after receiving CFA induction. Both the synovium and disc became thickened, opaque, and head width increased in the CFA-induced TMJ-OA group (marked in black circle) that recovered after treatment in hPRP/HA group. Each group n = 3 rats. *p < 0.05, **p < 0.01, and ***p < 0.001, the results are shown as mean ± SD for three replicates

Next, to investigate the therapeutic effect of combined hPRP/HA therapy in an in-vivo model, we first induced TMJ-OA rat through CFA administration at 0 and 14th day and then in the 21st day applied intra-articular injections of the hPRP/HA in the treatment group or PBS in the control group (Fig. 4A). Nociceptive responses were monitored through changes in body weight (Fig. 4B), which showed no significant difference. The head width increased in CFA-induced TMJ-OA was restored by hPRP/HA treatment down reaching to that of the control group at 4 weeks post-treatment (Fig. 4C).

### MRI of TMJ in a TMJ-OA rat after combined hPRP/HA therapy

**Fig. 5:**
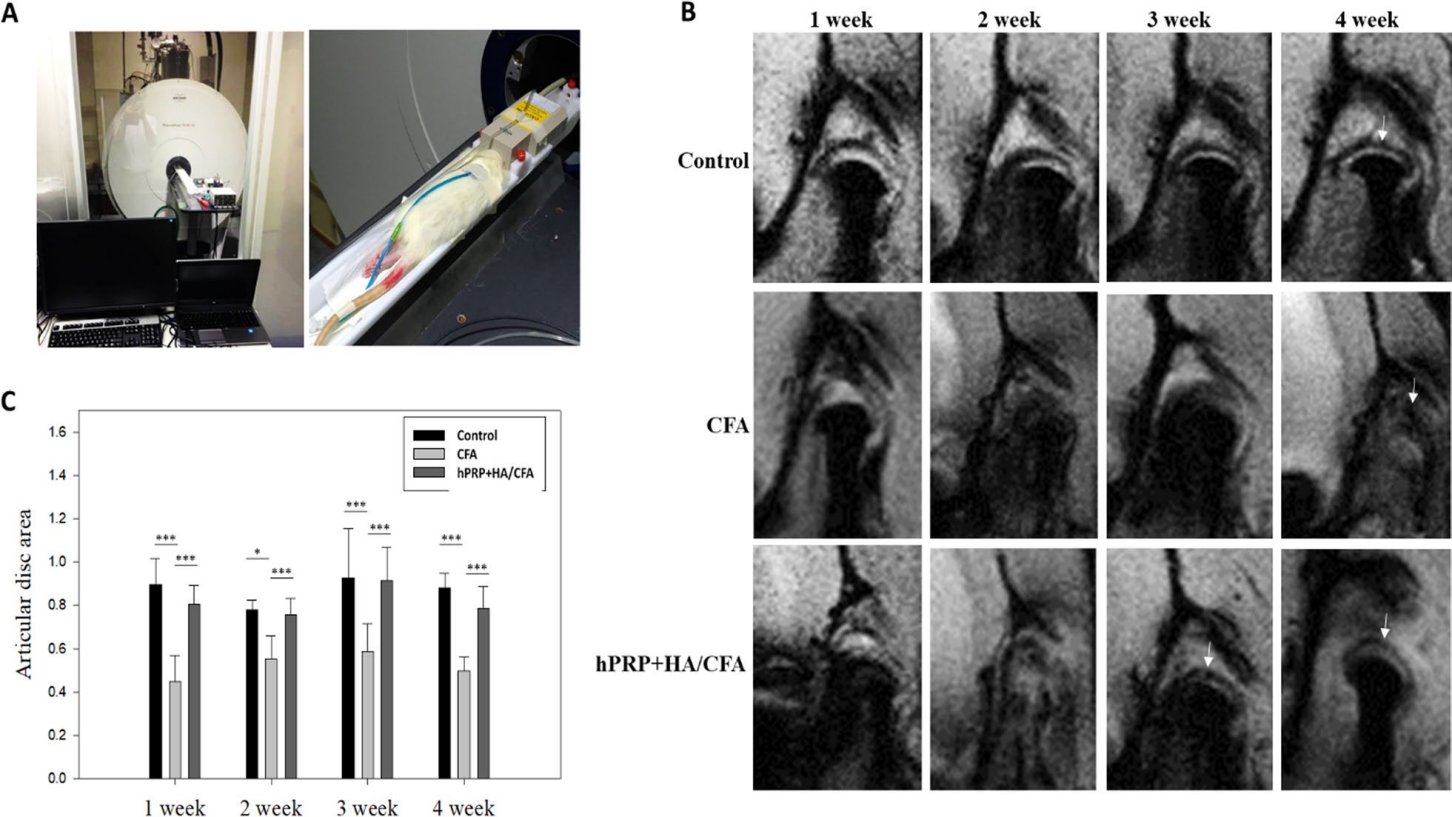
High magnetic field magnetic resonance imaging (MRI) applied to the TMJ in the rat. **A** Overview of the 7 T micro-imaging system. **B** MRI scan of control, CFA, and hPRP/HA-treated groups at 1–4 weeks posttreatment. **C** Measurement of the articular spaces in the coronal view. *p < 0.05, **p < 0.01, and ***p < 0.001 and the results are shown as mean ± SD for three replicates

The therapeutic potential of the hPRP/HA was then evaluated using MRI (Fig. 5A) before and after treatment. At the early point of 1 week, MRI data indicated that no significant difference was observed in the articular disc area, articular cartilage, and temporal bone between hPRP/HA-treated and untreated groups (Fig. 5B, first column). However, after 3 weeks of treatment, the hPRP/HA-treated group exhibited regeneration of both the articular disc area and temporal bone and reduced fibrous thickness of the articular cartilage compared with the CFA-only group. Moreover, after 4 weeks of treatment, compared with the CFA-only group, the condyle in the hPRP/HA-treated group showed complete improvement in the articular disc area, temporal bone, and articular cartilage, which was similar to that of the control group (Fig. 5B, last column). Quantification of the articular disc area also revealed an improvement in a time-dependent manner after treatment with hPRP/HA (Fig. 5C).

### Combined hPRP/HA therapy reverses TMJ degeneration in TMJ-OA

**Fig. 6:**
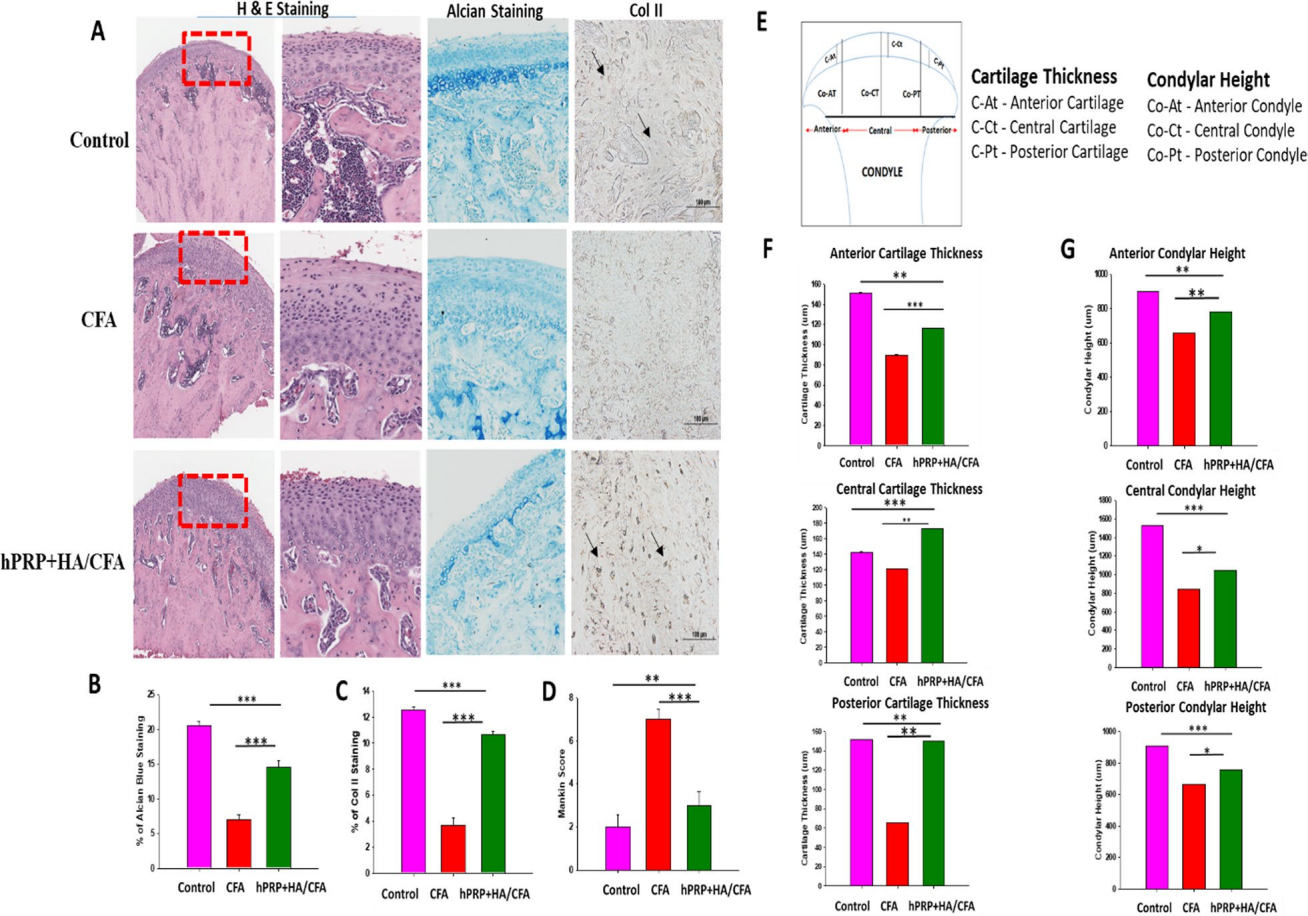
hPRP/HA promote TMJ repair and regeneration in OA. Histopathological evaluation indicated that hPRP/HA restored TMJ matrix synthesis in OA. **A** Hematoxylin and eosin (H&E), Alcian blue staining, and immunohistochemical staining for Col-II at 4 weeks (black arrow indicate the presence of Col-II). Representative images (*n* = 3). Scale bars: 500 or 100 μm. **B** Percentage areal deposition of proteoglycan by quantification of Alcian-stained area, and **C** percentages of Col-II cells in TMJ condylar cartilage. **D** Mankin scores of samples at 4 weeks. **E** Schematic of the anterior, central, and posterior regions of the rat TMJ condylar head. The condylar height was measured at each region, including co-At: anterior height; co-Ct: central height; and co–Pt: posterior height of condyle. The cartilage thickness was also measured at each region, including C-At: anterior thickness; C-Ct: central thickness; and C-Pt: posterior thickness of cartilage. Measurement of **F** cartilage thickness and **G** condylar height at the anterior, posterior, and central regions. *p < 0.05, **p < 0.01,, and ***p < 0.001 and the results are shown as mean ± SD for three replicates

At 4 weeks post-treatment, the hPRP/HA-treated group exhibited effective restoration of the TMJ condylar structure, with marked improvements in overall cartilage thickness, matrix deposition, cellularity, and condylar height that were comparable to those of the control group (Fig. 6). Compared with the CFA-only group, the hPRP/HA-treated group exhibited reduction in fibrous thickening of the cartilage and enhanced matrix restoration, with higher areal deposition of proteoglycan and Col-II, as revealed by H&E staining (Fig. 6A). Quantification through Alcian blue and Col-II staining showed that the hPRP/HA-treated group exhibited increased matrix and Col-II deposition by 15% and 10%, respectively (Fig. 6B, C). Mankin score was also evaluated for tissue injury. At 4 weeks post-treatment, the Mankin score of the CFA-only group increased deterioration level to 7.5, and the score of the hPRP/HA-treated group further reduced to 3.5, which is close to the Mankin score of the control group (2.0) (Fig. 6D). Moreover, the condyle and cartilage were divided into three main regions: anterior, central, and posterior to be examined (Fig. 6E). Compared with the CFA-only group, the hPRP/HA-treated group displayed increased cartilage thickness in the anterior region (116.4 ± 32 μm), central region (172.9 ± 30 μm), and posterior region (149 ± 42 μm) (Fig. 6F); as well as improvements in the condylar height in the anterior region (779.3 ± 25 µm), central region (1046.8 ± 40 µm), and posterior region (759.7 ± 15 µm) (Fig. 6G). Collectively, the results demonstrated that combined hPRP and HA treatment alleviated TMJ degeneration by suppressing inflammation and pain, reducing adverse fibrosis, and promoting overall matrix restoration of the TMJ condylar cartilage and subchondral bone in later stages of repair. Therefore, the combined hPRP/HA treatment could alleviate tissue injury and enhance tissue repair in TMJ-OA.

### hPRP/HA therapy alleviates subchondral bone deterioration in TMJ-OA

**Fig. 7:**
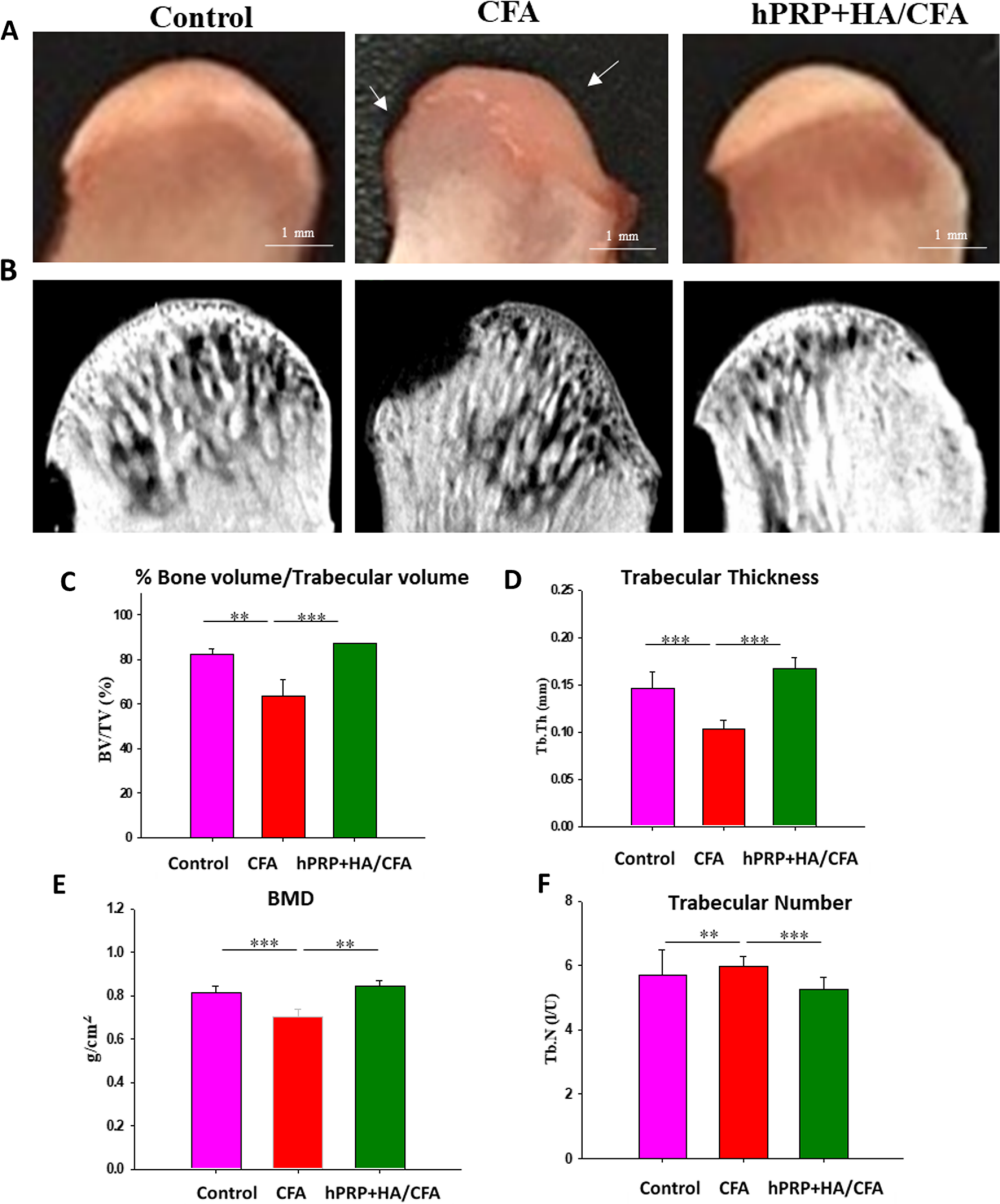
hPRP/HA treatment restored TMJ subchondral bone volume and architecture in OA. Rat TMJs were harvested for micro-CT analysis at 4 weeks posttreatment of hPRP/HA. **A** Macromorphological view (white arrow indicates damage area of subchondral bone) and **B** sagittal view. **C** BV/TV, **D** trabecular thickness, **E** BMD, and **E** trabecular number. Data represent mean ± SD. *p < 0.05, **p < 0.01, and ***p < 0.001 compared with control and CFA group and the results are shown as mean ± SD for three replicates. White arrow indicate damage area of TMJ

The overall reduction in the condylar height with decreased cartilage thickening in the CFA-only group suggested subchondral bone erosion (Fig. 7A). We therefore investigated bone structure changes, including macromorphology, percentage of bone volume (BV), trabecular volume (TV), trabecular number, trabecular thickness, and bone mineral density (BMD) through micro-CT analysis (Fig. 7B). Combined hPRP/HA treatment attenuated bone loss, as indicated by increased BV (Fig. 7C), trabecular thickness (Fig. 7D), and BMD (Fig. 7E). However, relative to the CFA-only group, the hPRP/HA-treated group exhibited a decreased trabecular number (Fig. 7F).

### Demographic and evaluation parameters for the clinical outcome of hPRP/HA treatment

**Fig. 8:**
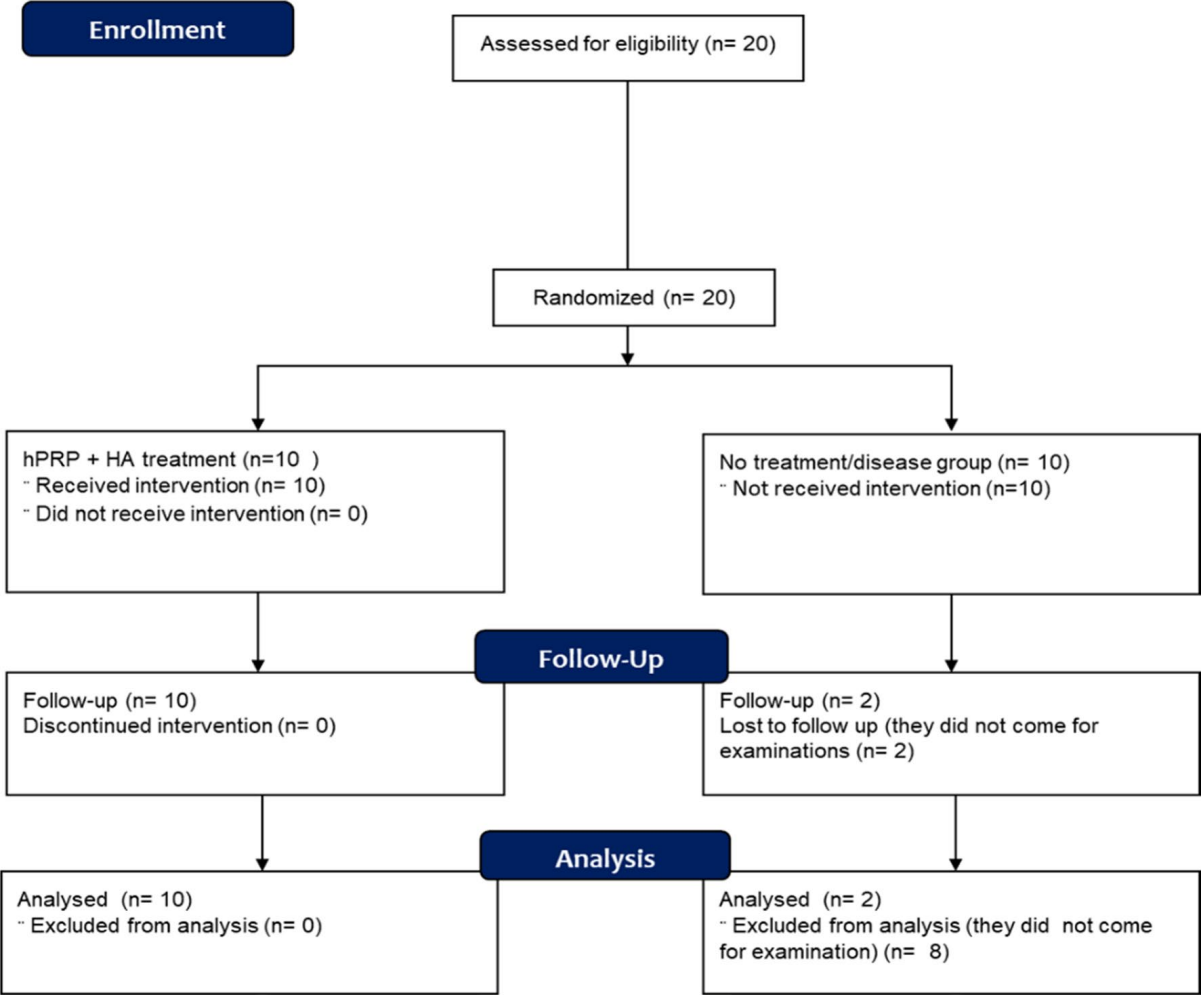
Consort flowchart for clinical trial of hPRP + HA in TMJ-OA disease

**Fig. 9:**
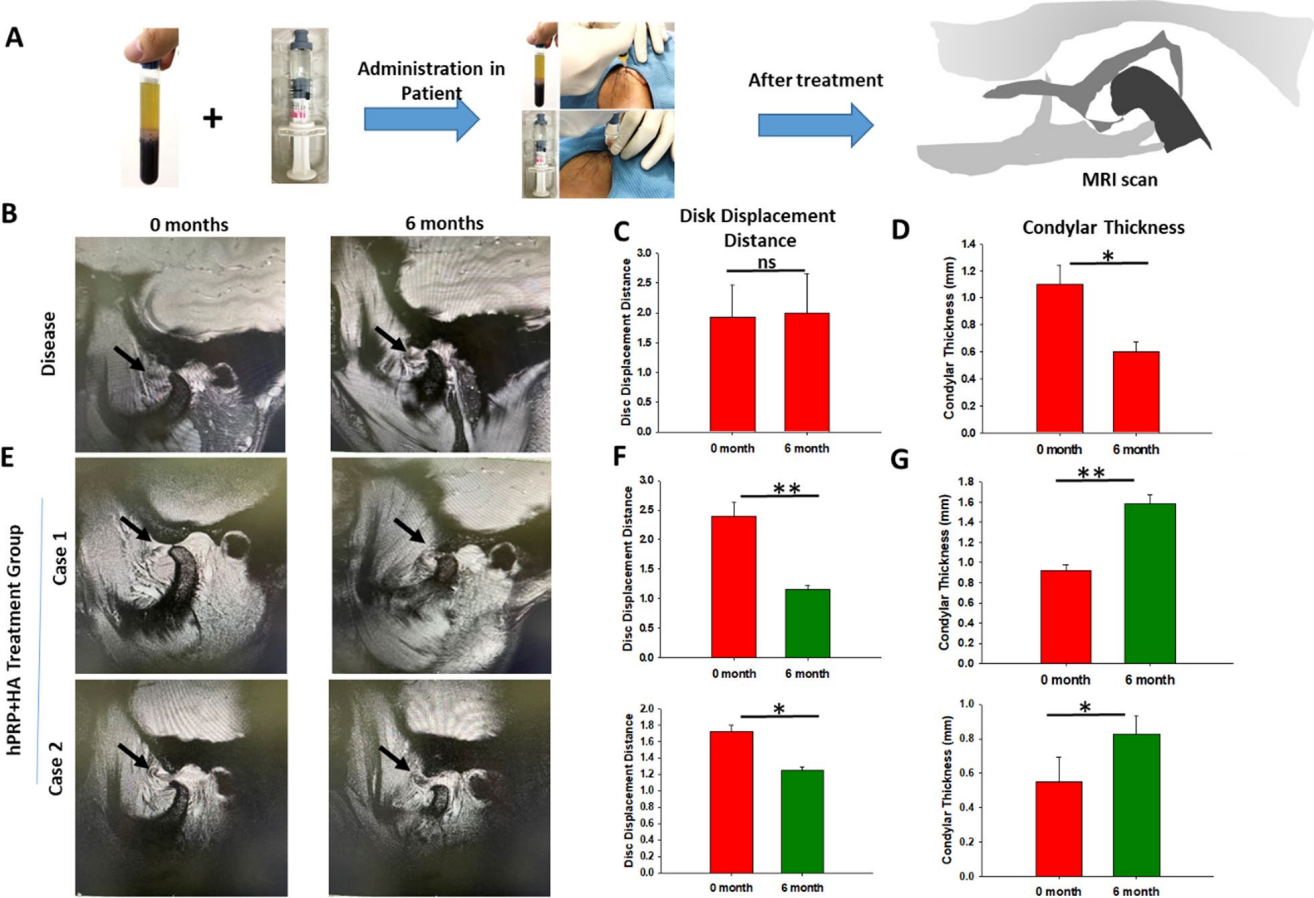
Therapeutic potential of the combine hPRP/HA treatment in the clinical study. **A** Schematic of HA and PRP administration into the superior space of TMJ-OA patients. **B** Disease group without treatment at 0 and 6 months of oblique sagittal MRI images represent disk displacement and osteoarthritis become worse. Quantification of the disc displacement distance from the condyle (**C**) and condylar thickness were measured at 0 and 6 months (cases 1 and 2, n = 1) (**D**). In the treatment group (**E**), patients of disc displacement with reduction (DDR), disc displacement without reduction (DDWR) are designated as case 1, and case 2, respectively, that were associated with disk degeneration, and osteoarthritis of the condyle at 6 months post-treatment. Oblique sagittal MRI images indicated disc recapture and condylar remodeling after using the hPRP/HA treatment compared with the disease or untreated group. In the treatment group disc displacement distance from the condyle (**F**) and condylar thickness (**G**) was measured at 0 and 6 months. *p < 0.05, **p < 0.01, and ***p < 0.001 compared with I + T and control group using paired t test

After the evaluation of combined hPRP/HA therapeutic effect on in-vitro and in-vivo studies, we performed a pilot clinical study in Taipei medical University hospital. In the human clinical trial, we enrolled 20 patients, which were randomly divided into treated group (10 patients) and placebo/non-treated/disease group (10 patients) (Fig. 8). No deaths occurred during the study, and no adverse events led to treatment discontinuation or study termination. TMJ-OA patient was administered with the hPRP/HA injections (Fig. 9A), the patients’ condition were evaluated using MRI, visual Analogue scale (VAS), and examination of Maximum Mouth Opening (MMO), and Activity of Daily life (ADL).

### MRI screening of the TMJ after hPRP/HA therapy in patients with TMJ-OA

MRI images were evaluated before and after treatment for disc displacement, disc degeneration, condylar bone changes as signs of bone degenerative process, or disk perforation. In case of disc displacement with reduction (DDR) in the closed mouth position, the posterior region of the disk is anterior to the condyle, whereas in the open mouth position, it returns to the normal position. However, in case of disc displacement without reduction (DDWR) in the closed mouth position, the posterior region of the disk is anterior to the condyle, and the disc size is normal, whereas in the open mouth position, the posterior region of the disk cannot return to its normal position between the condyle and articular eminence [24]. In the disease group/non-treated, MRI images at 0 and 6 months revealed that disc displacement (Fig. 9B) and its distance from the condyle remained unchanged (Fig. 9C); however, the condyle was degraded, and its thickness was reduced (Fig. 9D). By contrast, in the treatment group, after 6-month MRI data revealed slight restoration of disk displacement in DDR cases 1 and DDWR case 2 (Fig. 9E). Moreover, in both cases, disc displacement toward the condyle occurred (Fig. 9F), decreasing the disk displacement distance and increasing the condyle thickness to restore the shape of the condyle (Fig. 9G).

### Clinical assessment after hPRP/HA therapy in TMJ-OA patients.

**Fig. 10:**
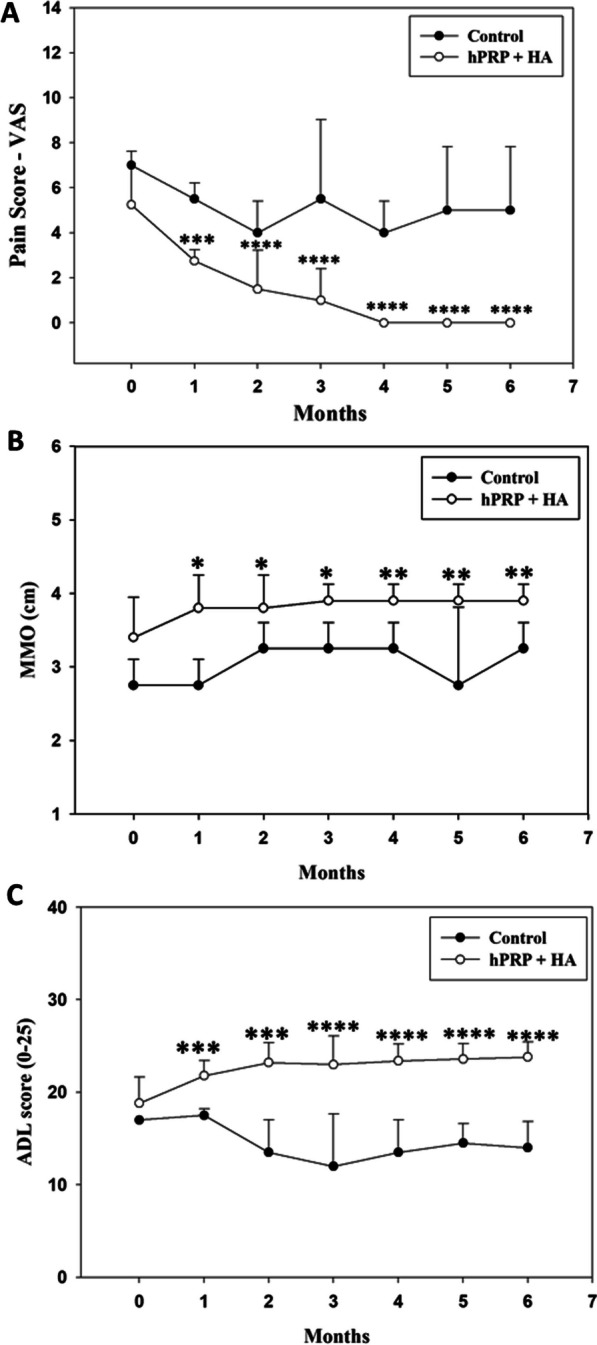
Clinical findings of hPRP/HA treatment. **A** Comparison of pain (VAS score) during the follow-up period with the preoperative level of pain. A greater decrease in pain was observed in the hPRP/HA-treated group. **B** Comparison of MMO during the follow-up period with preoperative MMO. A progressive increase in MMO was observed in the hPRP/HA-treated group. **C** Comparison of ADL during the follow-up period with the preoperative level of ADL was also increased in hPRP/HA-treated group. Clinically, the MMO and ADL scores were significantly increased in the hPRP/HA-treated group compared to the control (non-treated/disease) group, and the pain (VAS) score was significantly decreased. *p < 0.05, **p < 0.01, ***p < 0.001, and ****p < 0.0001 compared within hPRP + HA group using paired t test

After the treatment of hPRP–HA, patients were followed up to 6 month (Additional file 1: Table S1), and improvement in pain was assessed using VAS. VAS pain scores significantly decreased to 2.7 at 1 month and after 6 months the VAS pain score is 0 compared with 4.5 score in patients with TMJ-OA (Fig. 10A) (Table 2; Additional file 1: Tables S2, S3.1, and S3.2).

**Table 2:** VAS result for the control and hPRP + HA study group

Group	VAS score						
Month	0	1	2	3	4	5	6
Control	7(± 1.63)	5.5(± 1.2)	4(± 1.15)	5.5(± 1.2)	4.5(± 0.70)	4.5(± 0.70)	4.5(± 0.70)
HA/PRP	5.3(± 1.63)	2.7(± 0.91)	1.8(± 1.22)	1(± 1.04)	0.2(± 0.42)	0.1(± 0.31)	0(± 0)

Patients with TMJ-OA are also not able to open their mouth properly. Therefore, we also evaluated the MMO based on the parameters in Table 3 (Additional file 1: Tables S4, S5.1, and S5.2). MMO was recorded in cm, and results revealed a significant increase in MMO after treatment with hPRP/HA (Fig. 10B). Patients with TMJ-OA have pain, reduced MMO, and decreased quality of life, thus considerably reducing their ADL, such as eating, talking, and laughing. ADL were measured in terms of scores of 0–25 (Table 4; Additional file 1: Tables S6, S7.1, and S7.2). We observed that after the treatment with hPRP/HA, the ADL score increased as compared to patients with TMJ-OA (Fig. 10C).

**Table 3:** MMO result for the control and hPRP + HA study group

Group	MMO						
Month	0	1	2	3	4	5	6
Control	2.75(± 0.4)	2.75(± 0.4)	3.25(± 0.3)	3.25(± 0.3)	3.25(± 0.3)	2.75(± 1.0)	3.2(± 0.35)
hPRP/HA	3.4(± 0.39)	3.7(± 0.37)	3.8(± 0.27)	3.8(± 0.35)	3.9(± 0.21)	3.9(± 0.21)	3.9(± 0.21)

**Table 4:** ADL result for the control and hPRP + HA study group

Group	ADL						
month	0	1	2	3	4	5	6
Control	17.0(± 2.7)	14.5(± 0.7)	14.5(± 1.9)	12(± 3.6)	13.5(± 3.5)	14.5(± 2.1)	14.5(± 2.1)
hPRP/HA	18.8(± 2.2)	23.6(± 1.4)	23.8(± 1.6)	22.8(± 2.04)	23(± 1.3)	23.6(± 1.2)	23.6(± 1.2)

## Discussion

This study is the first to investigate the effect of combined hPRP/HA therapy on TMJ-OA from bench to bedside, including in-vitro (2D and 3D), animal TMJ-OA models, and human clinical trial; our results indicated a reduction in inflammation, tissue degeneration, and pain, followed by increased matrix synthesis to regenerate the TMJ tissue.

In TMJ-OA pathogenesis, adverse inflammation activates catabolic matrix degradation that induces condyle cartilage degeneration, apoptosis, necroptosis, and chondrocyte death to exacerbate joint damage, pain, and disease progression [2]. Various proinflammatory molecules, including IL-1β (I) and TNF-α (T), have been reported to play crucial roles in TMJ-OA progression [25]. Their levels are significantly elevated in the synovial fluid of patients with TMJ-OA, and they are believed to mediate condyle cartilage degeneration and matrix degradation by inducing MMP3 and MMP13 expression [2, 26]. Commonly, the elevated inflammation reduces chondrocyte proliferation and induces alterations in the condyle cartilage matrix due to apoptosis and necroptosis [26]. IL-1β enhances calcium influx in chondrocytes, which inhibits mitochondrial function and leads to chondrocyte apoptosis [27]. TNF-α can facilitate apoptosis and necroptosis by activating the death receptor pathway, NF-kβ and generating oxidative stress, respectively, to exacerbate cartilage degradation [28]. Therefore, to evaluate the therapeutic potential of the hPRP/HA combination, we used I + T to create in-vitro TMJ OA disease models. In this study, after hPRP/HA clinical trial in dish, our data indicated that hPRP/HA treatment mainly targeted the essential pathological features of TMJ-OA inflammation, proliferation, and matrix degradation. hPRP/HA treatment ameliorated inflammation in the in-vitro TMJ-OA model, as evidenced by the increased proliferation and reduced gene expression of inflammatory molecules, leading to enhanced chondrogenesis. It is also consistent with the IHC staining of proteoglycan and Col-II, demonstrating that hPRP/HA treatment increased chondrogenesis differentiation for matrix accumulation.

We also investigated the therapeutic potential of the hPRP/HA treatment in an in-vivo mimic microenvironment using a 3D rotary cell culture system. The rotary cell culture system is one of several fluid dynamic culture systems, and it has been widely used in 3D cell cultures [29]. Our previous study demonstrated a 3D neocartilage model to mimic a similar microenvironment of in-vivo knee OA [21]. In this study, TMJ rat chondrocytes were grown in a 3D collagen construct that resembled the condyle, and TMJ-OA was induced through proinflammatory molecules (I + T) that deformed the macromorphology, reduced condylar height, degraded the outer surface of cartilage, and promoted proteoglycan deposition. We observed that after treatment with hPRP/HA, the 3D condyle macromorphology was reformed with increases in the condylar height. After treatment, Col-II and proteoglycan deposition indicated the promotion of chondrocyte regeneration and matrix restoration of the condylar cartilage. Overall, our data showed that the clinical effect of hPRP/HA treatment in-vitro or in-vivo mimic overcomes proinflammatory-induced TMJ-OA through chondrocyte recovery, matrix restoration, and condyle cartilage regeneration.

To elucidate the mechanism underlying the effect of the hPRP/HA on matrix restoration, proliferation, and proinflammatory activity, we analyzed gene expression after hPRP/HA treatment to identify which genes could elicit some of the observed hPRP/HA effects. Studies have reported that combined hPRP/HA therapy upregulates the expression of SOX9, an essential element in initiating chondrogenesis and maintaining the matrix of the condyle cartilage [30]. Our results indicated that SOX9 played a role in osteochondral repair by inhibiting IL-1β and COX2, which reduced chondrocyte proliferation, but the proliferation was then enhanced by hPRP/HA treatment. SOX9 gene knock out mice showed that chondrocytes proliferation and articular cartilage regeneration were inhibited, and bone morphogenetic protein levels was decreased compared with normal mice [31]. Sox9 mainly binds to chondrocyte-specific enhancers such as *Col-II* and *Col-I,* which are essential for cartilage formation [32]. In this study, we also evaluated *Col-I* and *Col-II* gene expression because these genes have been implicated in the maintenance of chondrocyte matrix homeostasis: *Col-I* is mainly responsible for hypertrophic chondrocytes that secrete fibrocartilage to induce fibrosis, whereas *Col-II* mainly helps to maintain the matrix of the cartilage [33, 34]. *Col-I* gene expression was increased to reduce *Col-II* expression in the I + T-induced in-vitro TMJ-OA model, and changes in gene expression have been reported to accelerate OA development in animal models [32]. Because hPRP/HA treatment upregulated the gene expression of Col-II and inhibited *Col-I* expression, we postulate that SOX9 activation contributes to repair in TMJ-OA by hPRP/HA through inhibiting hypertrophic chondrocytes and matrix degradation. The in-vitro TMJ-OA model also revealed that hPRP/HA increased proteoglycan synthesis by upregulating the aggrecan gene and inhibiting MMP3, MMP13, and COX2 in I + T-treated chondrocytes.

Following the in-vitro experiments showing the therapeutic potential of the hPRP/HA in TMJ-OA, we evaluated the effects of the hPRP/HA in TMJ-OA animal model. A previous study also showed effect of hPRP and HA on the regeneration of defects in the articular disc of TMJ in a rabbit model [18]. However, in our study we induced an inflammatory condition by using a chemical mediator CFA to establish a TMJ-OA animal model. Inflammation-induced OA models have distinct molecular pathophysiology that promotes the translation of disease-modifying OA therapies from animal models to human clinical trials [35]. The CFA-induced model has demonstrated similarities to human TMJ-OA pathology and has been widely used for pathophysiological and morphological analyses [36]. CFA was used as a chemical mediator, and its intra-articular injection in rats initiates inflammation by activating IL-1β and TNF-α, leading to cartilage degradation [37]. Recently, a study of ankle joint mobilization in a mouse model also used CFA to induce IL-1β and TNF-α expression [38]. MRI revealed that CFA-induced TMJ-OA caused condyle degeneration, articular disc area reduction, and disc degradation, all of which were ameliorated after 4 weeks of hPRP/HA treatment. In TMJ-OA the subchondral bone is also a key element in disease progression. A previous study reported that spontaneous abnormalities in the condyle subchondral bone can induce progressive cartilage degradation in mice [4]. We observed that hPRP/HA treatment restored the subchondral bone by increasing the BMD and trabecular thickness to prevent cartilage degradation. In TMJ-OA rats, hPRP/HA treatment also increased *Col-II* and proteoglycan synthesis to restore the degraded matrix and condyles, which correlated with the improvement in the Mankin score for TMJ-OA degenerative changes.

The clinical trial in dish (in vitro, 3D culture), and animal trials indicated that combined hPRP/HA therapy effectively promotes chondrocyte proliferation and improves cartilage repair. Therefore, we also evaluated the therapeutic potential of the hPRP/HA in human clinical trials. Most studies have applied the PRP or HA, and PRP/HA combination in the treatment of knee OA and have indicated that the PRP/HA provides better clinical improvement in terms of symptoms and function [39, 40]. In this study, we used hPRP/HA treatment in human clinical trial and enrolled 20 patients with TMJ OA that divided into a treatment (n = 10) and a control (no-treatment/disease group) group (n = 10) for follow-up to 6 months, but from which 8 participants of control group dropped out due to no effect during trial. Although we lost to follow up 80% of the patient of control (placebo) group, however, missing data is not as problematic in interpreting the results of a trial with statistically significant effect estimates [41, 42]. Moreover, the patients lost to follow-up were not statistically significantly associated with treatment effect, if the reason for dropouts and percentage of dropout patients were provided [43]. The information required to assess the loss to follow-up and attribution of events in accordance with randomization was clearly reported in consort flowchart (Fig. 8). MRI scanning was used to evaluate disc displacement**/**reduction**,** disc degeneration/deformation, and condyle bone changes in patients with TMJ-OA. We observed that after 6 months of hPRP/HA treatment, patients with DDR exhibited reduced changes in the disc, whereas in some patients with DDWR, joint effusion was ameliorated, and the mandibular condyle was regenerated. A previous study also reported that after 6 months of HA treatment, patients with DDR exhibited reduced pain and TMJ noise only [14]. In a clinical trial of pain reduction in patients with TMJ OA, at 6 months after PRP injection, VAS pain scores decreased to a moderate level in patients with TMJ-OA [44]. HA reduces inflammatory molecules such as metalloproteinase enzymes, prostaglandins, and oxidative stress from the synovial fluid, and hPRP inhibits inflammatory molecules at the injury site [44, 45]. The exclusion of inflammatory molecules from the fluid could result from injecting HA and hPRP. Our findings indicated that hPRP/HA treatment could not only reduce pain and but increase MMO and ADL at 6 months of follow-up. The limitation of our study was that the sample size in one group were decreased due to the drop out cases. Therefore, we performed the analysis based on per protocol method and excluded the all drop out data of participant, according to CONSORT principle. This study did not evaluate the feasibility of non-treated and hPRP/HA-treated in a clinical setting; rather, the study was designed to confirm that hPRP/HA combined therapy have the significant therapeutic effects for TMJ-OA not only to ameliorate the pain but also regenerate the condyle, and reduce the disc displacement. Clinically, these outcomes clearly demonstrated the much more efficacy of hPRP/HA treatment in patients with TMJ-OA. We have observed a wide spectrum of effects related to tissue repair and regeneration in the present study, such as the modulation of cellular communication, cellular structure, tissue metabolism, and immune modulation. Overall, our study demonstrated that intra-articular hPRP/HA therapy effectively controls pain and stimulates tissue repair in TMJ-OA.

## Conclusion

**Fig. 11:**
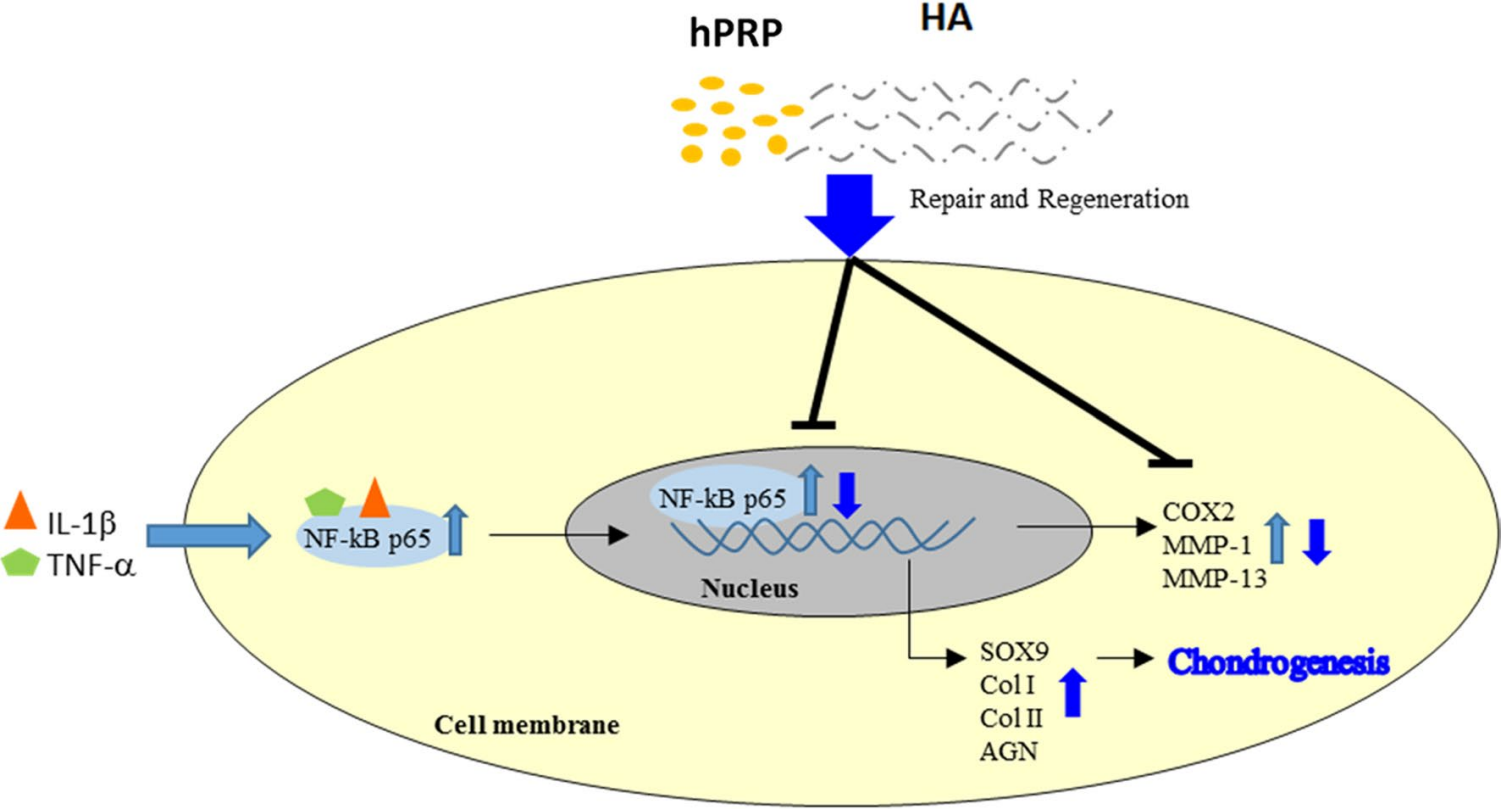
Schematic of therapeutic mechanism action of hPRP/HA therapy in TMJ-OA disease. hPRP (human platelet rich plasma) and HA (hyaluronic acid) ameliorates the bone degradation by upregulation chondrogenic genes in inflammatory (IL-1β and TNF-α) induced TMJ-OA model

TMJ OA is a degenerative joint disease characterized by chronic pain, cartilage degradation, and subchondral bone erosion, and it is caused by perturbation of joint homeostasis, resulting in a net loss of cells and matrix. Here, we demonstrated that the hPRP/HA clinical trial in dish, animal, and human TMJ-OA could reduce pain and repair osteoarthritic TMJs by mounting a well-coordinated response of attenuating inflammation, enhancing proliferation and matrix synthesis, and promoting TMJ repair and regeneration (Fig. 11). Therefore, hPRP/HA treatment is a potential disease-modifying therapy for TMJ-OA.

### Supplementary Information

**Additional file 1: Figure S1.** The effect of HA (250 μg/mL) or hPRP (1 ng/mL), and hPRP + HA (1 ng/mL + 250 μg/mL) on the cell numbers of rat TMJ chondrocytes after 2-day treatment with IL1β + TNF-α (I + T)–conditioned medium. I (10 ng/mL) + T (20 ng/mL) were added to the medium to create an in-vitro proinflammatory cytokine–induced arthritic cell model. **Table S1.** Detailed number of Patients participated in Clinical trial and their drop out months. **Table S2.** Intergroup comparison VAS results for the control and hPRP/HA study groups. **Table S3.2.** Intragroup VAS results for the within hPRP/HA study groups. **Table S4.** Intergroup comparison MMO results for the control and hPRP/HA study groups. **Table S5.1.** Intragroup MMO results for the within control groups. **Table S5.2.** Intragroup MMO results for the within hPRP/HA groups. **Table S6.** Intergroup comparison ADL results for the control and hPRP/HA study groups. **Table S7.1.** Intragroup ADL results for the within control groups. **Table S7.2.** Intragroup ADL results for the within hPRP/HA groups.

## Data Availability

All data analysed or generated during the study are included in this published article.
