# Supplementary material for: Platelet-derived biomaterial with hyaluronic acid alleviates temporal-mandibular joint osteoarthritis: clinical trial from dish to human
Source: J Biomed Sci. 2023 Sep 11;30:77. doi: 10.1186/s12929-023-00962-y (PMC10494357; doi:10.1186/s12929-023-00962-y)
Supplement: Supplementary file 1 — Additional file 1: Figure S1. The effect of HA (250 μg/mL) or hPRP (1 ng/mL), and hPRP + HA (1 ng/mL + 250 μg/mL) on the cell numbers of rat TMJ chondrocytes after 2-day treatment with IL1β + TNF-α (I + T)–conditioned medium. I (10 ng/mL) + T (20 ng/mL) were added to the medium to create an in-vitro proinflammatory cytokine–induced arthritic cell model. Table S1. Detailed number of Patients participated in Clinical trial and their drop out months. Table S2. Intergroup comparison VAS results for the control and hPRP/HA study groups. Table S3.2. Intragroup VAS results for the within hPRP/HA study groups. Table S4. Intergroup comparison MMO results for the control and hPRP/HA study groups. Table S5.1. Intragroup MMO results for the within control groups. Table S5.2. Intragroup MMO results for the within hPRP/HA groups. Table S6. Intergroup comparison ADL results for the control and hPRP/HA study groups. Table S7.1. Intragroup ADL results for the within control groups. Table S7.2. Intragroup ADL results for the within hPRP/HA groups. [file 12929_2023_962_MOESM1_ESM.docx]

**ADDITIONAL INFORMATION**

figure S1:


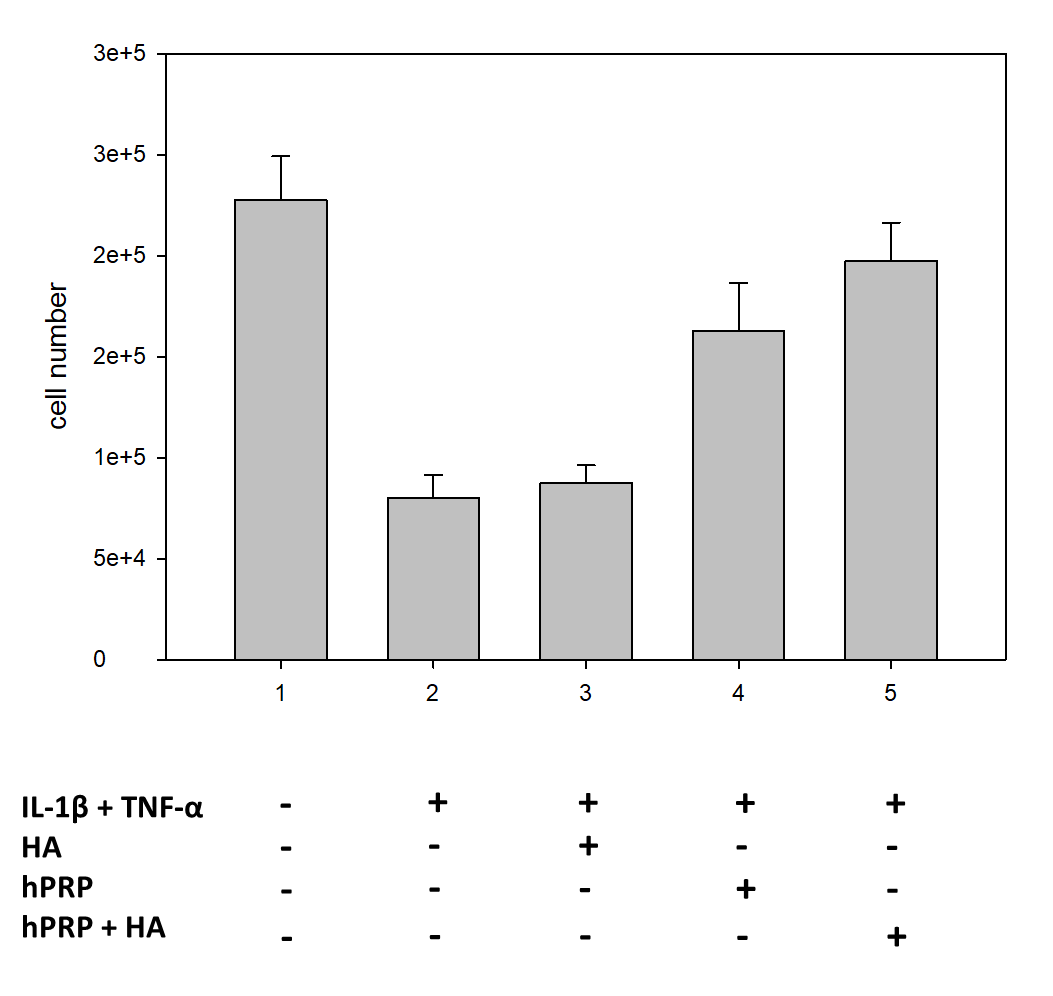


**figure S1:** The effect of HA (250 μg/mL) or hPRP (1 ng/mL), and hPRP + HA (1 ng/mL + 250 μg/mL) on the cell numbers of rat TMJ chondrocytes after 2-day treatment with IL1β + TNF-α (I + T)–conditioned medium. I (10 ng/mL) + T (20 ng/mL) were added to the medium to create an *in-vitro* proinflammatory cytokine–induced arthritic cell model.

**Table S1: Detailed number of Patients participated in Clinical trial and their drop out months**

| **Months** | **Number of patients** | | **Drop out** | |
| --- | --- | --- | --- | --- |
|  | **Ctrl** | **hPRP + HA** | **Ctrl** | **hPRP + HA** |
| 0M | 10 | 10 | 0 | 0 |
| 1M | 7 | 10 | 3 | 0 |
| 2M | 5 | 10 | 2 | 0 |
| 3M | 3 | 10 | 2 | 0 |
| 4M | 2 | 10 | 1 | 0 |
| 5M | 2 | 10 | 0 | 0 |
| 6M | 2 | 10 | 0 | 0 |

*Drop out due to lack of improvement

**Table S2: Intergroup comparison VAS results for the control and hPRP/HA study groups.**

| **Months** | **Number of patients** | | **Mean + SD** | | **p-value** |
| --- | --- | --- | --- | --- | --- |
|  | **Ctrl** | **HA+PRP** | **Ctrl** | **HA+PRP** | **Treatment group compared with Ctrl** |
| 0M | 2 | 10 | 7 (±1.639) | 5.3 (±1.63) | 0.004725 |
| 1M | 2 | 10 | 5.57(±1.2) | 2.7 (±0.91) | 0.027051 |
| 2M | 2 | 10 | 4 (±1.15) | 1.8 (±1.22) | 0.119051 |
| 3M | 2 | 10 | 5.5 (±1.29) | 1 (±1.04) | 0.008199 |
| 4M | 2 | 10 | 4.5 (±0.707) | 0.2 (±0.421) | 0.028903 |
| 5M | 2 | 10 | 4.5 (±0.707) | 0.1 (±0.316) | 0.031346 |
| 6M | 2 | 10 | 4.5 (±0.707) | 0 | 0.031346 |
| **LOCF** |  |  |  |  |  |
| 0M | 10 | 10 | 6.3 (±1.56) | 5.3 (±1.63) | 0.07909771 |
| 1M | 10 | 10 | 6.1(±1.37) | 2.7 (±0.91) | 0.000153511 |
| 2M | 10 | 10 | 5.8 (±1.54) | 1.8 (±1.22) | 0.000121062 |
| 3M | 10 | 10 | 6.2 (±1.39) | 1 (±1.04) | 1.57423E-06 |
| 4M | 10 | 10 | 5.9 (±1.52) | 0.2 (±0.421) | 7.9572E-07 |
| 5M | 10 | 10 | 6 (±1.69) | 0.1 (±0.316) | 6.82609E-07 |
| 6M | 10 | 10 | 6 (±1.69) | 0 | 7.10911E-07 |

*Results from a two-sample t-test assuming unequal variances. LOCF: Last observation carry forward.

**Table S3.1: Intragroup VAS results for the within control groups.**

| **Months** | **Number of patients** | | | **Mean + SD** | | **p-value** | |
| --- | --- | --- | --- | --- | --- | --- | --- |
|  | | **Ctrl** | **Ctrl** | | **Within Ctrl group**  **(baseline 0 month)** | |  |
| 0M | | 2 | 7 (±1.639) | | ---- | |  |
| 1M | | 2 | 5.57(±1.2) | | 0.017945 | |  |
| 2M | | 2 | 4 (±1.15) | | 0.012194 | |  |
| 3M | | 2 | 5.5 (±1.29) | | 0.214368 | |  |
| 4M | | 2 | 4.5 (±0.707) | | 0.037771 | |  |
| 5M | | 2 | 4.5 (±0.707) | | 0.095842 | |  |
| 6M | | 2 | 4.5 (±0.707) | | 0.095842 | |  |
| **LOCF** | |  |  | |  | |  |
| 0M | | 10 | 6.3 (±1.56) | | ---- | |  |
| 1M | | 10 | 6.1(±1.37) | | 0.382373849 | |  |
| 2M | | 10 | 5.8 (±1.54) | | 0.241122825 | |  |
| 3M | | 10 | 6.2 (±1.39) | | 0.440996384 | |  |
| 4M | | 10 | 5.9 (±1.52) | | 0.28498421 | |  |
| 5M | | 10 | 6 (±1.69) | | 0.343190886 | |  |
| 6M | | 10 | 6 (±1.69) | | 0.343190886 | |  |

**Table S3.2: Intragroup VAS results for the within hPRP/HA study groups.**

| **Months** | **Number of patients** | | **Mean + SD** | | **p-value** | |
| --- | --- | --- | --- | --- | --- | --- |
|  | **hPRP/HA** | **hPRP/HA** | | **Within hPRP/HA group**  **(baseline 0 month)** | |  |
| 0M | 10 | 5.3 (±1.63) | | ------ | |  |
| 1M | 10 | 2.7 (±0.91) | | 0.000585 | |  |
| 2M | 10 | 1.8 (±1.22) | | 0.000123 | |  |
| 3M | 10 | 1 (±1.04) | | 7.98E-07 | |  |
| 4M | 10 | 0.2 (±0.421) | | 1.3E-05 | |  |
| 5M | 10 | 0.1 (±0.316) | | 5.49E-09 | |  |
| 6M | 10 | 0 | | 5.49E-09 | |  |

*Results from a two-sample t-test assuming equal variances within each groups.

**Table S4: Intergroup comparison MMO results for the control and hPRP/HA study groups.**

| **Months** | **Number of patients** | | **Mean + SD** | | **P-value** |
| --- | --- | --- | --- | --- | --- |
|  | **Ctrl** | **hPRP/HA** | **Ctrl** | **hPRP/HA** | **Treatment group compared with Ctrl** |
| 0M | 2 | 10 | 2.75 (±0.4) | 3.4 (±0.39) | 0.090561 |
| 1M | 2 | 10 | 2.756 (±0.47) | 3.75 (±0.37) | 0.047716 |
| 2M | 2 | 10 | 3.25 (±0.35) | 3.8 (±0.27 | 0.112938 |
| 3M | 2 | 10 | 3.25 (±0.35) | 3.87 (±0.35) | 0.115443 |
| 4M | 2 | 10 | 3.25 (±0.35) | 3.9 (±0.21) | 0.012849 |
| 5M | 2 | 10 | 2.75 (±1.06) | 3.9 (±0.21) | 0.022328 |
| 6M | 2 | 10 | 3.25 (±0.35) | 3.9 (±0.21) | 0.015549 |
| **LOCF** |  |  |  |  |  |
| 0M | 10 | 10 | 2.28 (±0.38) | 3.4 (±0.39) | 0.000361316 |
| 1M | 10 | 10 | 2.535 (±0.63) | 3.75 (±0.37) | 0.000143418 |
| 2M | 10 | 10 | 2.645 (±0.69) | 3.8 (±0.27 | 0.001073028 |
| 3M | 10 | 10 | 2.661 (±0.70) | 3.87 (±0.35) | 0.00136119 |
| 4M | 10 | 10 | 2.659 (±0.69) | 3.9 (±0.21) | 0.000391727 |
| 5M | 10 | 10 | 2.563 (±0.63) | 3.9 (±0.21) | 0.000111885 |
| 6M | 10 | 10 | 2.643 (±0.68) | 3.9 (±0.21) | 0.000320895 |

*Results from a two-sample t-test assuming unequal variances.

**Table S5.1: Intragroup MMO results for the within control groups.**

| **Months** | **Number of patients** | | **Mean + SD** | | **p-value** | |
| --- | --- | --- | --- | --- | --- | --- |
|  | **Ctrl** | **Ctrl** | | **Within Ctrl group**  **(baseline 0 month)** | |  |
| 0M | 2 | 2.75 (±0.4) | | ------- | |  |
| 1M | 2 | 2.756 (±0.47) | | 0.372772 | |  |
| 2M | 2 | 3.25 (±0.35) | | 0.035762 | |  |
| 3M | 2 | 3.25 (±0.35) | | 0.03391 | |  |
| 4M | 2 | 3.25 (±0.35) | | 0.032029 | |  |
| 5M | 2 | 2.75 (±1.06) | | 0.384132 | |  |
| 6M | 2 | 3.25 (±0.35) | | 0.059257 | |  |
| **LOCF** |  |  | |  | |  |
| 0M | 10 | 2.28 (±0.38) | | ---- | |  |
| 1M | 10 | 2.535 (±0.63) | | 0.382373849 | |  |
| 2M | 10 | 2.645 (±0.69) | | 0.241122825 | |  |
| 3M | 10 | 2.661 (±0.70) | | 0.440996384 | |  |
| 4M | 10 | 2.659 (±0.69) | | 0.28498421 | |  |
| 5M | 10 | 2.563 (±0.63) | | 0.343190886 | |  |
| 6M | 10 | 2.643 (±0.68) | | 0.343190886 | |  |

**Table S5.2: Intragroup MMO results for the within hPRP/HA groups.**

| **Months** | **Number of patients** | | **Mean + SD** | | **p-value** | |
| --- | --- | --- | --- | --- | --- | --- |
|  | **hPRP/HA** | **hPRP/HA** | | **Within hPRP/HA group**  **(baseline 0 month)** | |  |
| 0M | 10 | 3.4 (±0.39) | | ------- | |  |
| 1M | 10 | 3.75 (±0.37) | | 0.037447 | |  |
| 2M | 10 | 3.8 (±0.27 | | 0.032271 | |  |
| 3M | 10 | 3.87 (±0.35) | | 0.013713 | |  |
| 4M | 10 | 3.9 (±0.21) | | 0.001181 | |  |
| 5M | 10 | 3.9 (±0.21) | | 0.001181 | |  |
| 6M | 10 | 3.9 (±0.21) | | 0.001181 | |  |

*Results from a two-sample t-test assuming equal variances within each groups.

**Table S6: Intergroup comparison ADL results for the control and hPRP/HA study groups.**

| **Months** | **Number of patients** | | **Mean + SD** | | **P-value** |
| --- | --- | --- | --- | --- | --- |
|  | **Ctrl** | **hPRP/HA** | **Ctrl** | **hPRP/HA** | **Treatment group compared with Ctrl** |
| 0M | 2 | 10 | 17(±2.7) | 18.8 (±2.2) | 0.017637 |
| 1M | 2 | 10 | 14.5(±0.7) | 23.6 (±1.4) | 0.003254 |
| 2M | 2 | 10 | 14.5(±1.9) | 23.8 (±1.6) | 0.074132 |
| 3M | 2 | 10 | 12(±3.6) | 22.8(±2.04) | 0.109142 |
| 4M | 2 | 10 | 13.5(±3.5) | 23 (±1.3) | 0.076139 |
| 5M | 2 | 10 | 14.5(±2.1) | 23.6 (±1.2) | 0.042847 |
| 6M | 2 | 10 | 14.5(±2.1) | 23.6 (±1.2) | 0.042267 |
| **LOCF** |  |  |  |  |  |
| 0M | 10 | 10 | 14.9 (±2.1) | 18.8 (±2.2) | 0.004928717 |
| 1M | 10 | 10 | 15.3 (±2.2) | 23.6 (±1.4) | 3.64229E-05 |
| 2M | 10 | 10 | 14.9 (±2.4) | 23.8 (±1.6) | 5.13827E-06 |
| 3M | 10 | 10 | 14.7 (±3.2) | 22.8(±2.04) | 4.34501E-05 |
| 4M | 10 | 10 | 15.0 (±2.6) | 23 (±1.3) | 6.74492E-06 |
| 5M | 10 | 10 | 15.2 (±2.3) | 23.6 (±1.2) | 6.7848E-07 |
| 6M | 10 | 10 | 15.1 (±2.4) | 23.6 (±1.2) | 5.80126E-07 |

*Results from a two-sample t-test assuming unequal variances.

**Table S7.1: Intragroup ADL results for the within control groups.**

| **Months** | **Number of patients** | | **Mean + SD** | | **p-value** | |
| --- | --- | --- | --- | --- | --- | --- |
|  | **Ctrl** | **Ctrl** | | **Within Ctrl group**  **(baseline 0 month)** | |  |
| 0M | 2 | 17(±2.7) | | ------- | |  |
| 1M | 2 | 14.5(±0.7) | | 0.211325 | |  |
| 2M | 2 | 14.5(±1.9) | | 0.148237 | |  |
| 3M | 2 | 12(±3.6) | | 0.168867 | |  |
| 4M | 2 | 13.5(±3.5) | | 0.148237 | |  |
| 5M | 2 | 14.5(±2.1) | | 0.118754 | |  |
| 6M | 2 | 14.5(±2.1) | | 0.136197 | |  |
| **LOCF** |  |  | |  | |  |
| 0M | 10 | 14.9 (±2.1) | | ---- | |  |
| 1M | 10 | 15.3 (±2.2) | | 0.018393749 | |  |
| 2M | 10 | 14.9 (±2.4) | | 0.5 | |  |
| 3M | 10 | 14.7 (±3.2) | | 0.4252215 | |  |
| 4M | 10 | 15.0 (±2.6) | | 0.448570322 | |  |
| 5M | 10 | 15.2 (±2.3) | | 0.30822342 | |  |
| 6M | 10 | 15.1 (±2.4) | | 0.3849375 | |  |

**Table S7.2: Intragroup ADL results for the within hPRP/HA groups.**

| **Months** | **Number of patients** | | **Mean + SD** | | **p-value** | |
| --- | --- | --- | --- | --- | --- | --- |
|  | **hPRP/HA** | **hPRP/HA** | | **Within hPRP/HA group**  **(baseline 0 month)** | |  |
| 0M | 10 | 18.8 (±2.2) | | -------- | |  |
| 1M | 10 | 23.6 (±1.4) | | 0.001360863 | |  |
| 2M | 10 | 23.8 (±1.6) | | 5.20802E-05 | |  |
| 3M | 10 | 22.8(±2.04) | | 0.000327889 | |  |
| 4M | 10 | 23 (±1.3) | | 2.70694E-05 | |  |
| 5M | 10 | 23.6 (±1.2) | | 8.8115E-06 | |  |
| 6M | 10 | 23.6 (±1.2) | | 4.9502E-06 | |  |

*Results from a two-sample t-test assuming equal variances within each groups.
